# Broad‐Spectrum Engineered Multivalent Nanobodies Against SARS‐CoV‐1/2

**DOI:** 10.1002/advs.202402975

**Published:** 2024-10-07

**Authors:** Zhihong Wang, Zhuangzhuang Shi, Xiaochen Liao, Guiqi Quan, Hui Dong, Pinnan Zhao, Yangyihua Zhou, Ning Shi, Jie Wang, Yahui Wu, Chunxia Qiao, Xin ying Li, Ran Zhang, Zekun Wang, Tiecheng Wang, Xiang Gao, Jiannan Feng, Longlong Luo

**Affiliations:** ^1^ State Key Laboratory of Toxicology and Medical Countermeasures Beijing Institute of Pharmacology and Toxicology Beijing 100850 P. R. China; ^2^ Key Laboratory of Jilin Province for Zoonosis Prevention and Control Changchun Veterinary Research Institute Chinese Academy of Agricultural Sciences Changchun 130122 P. R. China; ^3^ Joint National Laboratory for Antibody Drug Engineering the First Affiliated Hospital, Henan University Kaifeng City Henan 475004 P. R. China; ^4^ Hunan Normal University School of medicine Changsha Hunan 410200 P. R. China

**Keywords:** antibody engineering, de novo design, multivalent nanobodies, nanobody, SARS‐CoV‐1, SARS‐CoV‐2

## Abstract

SARS‐CoV‐2 Omicron sublineages escape most preclinical/clinical neutralizing antibodies in development, suggesting that previously employed antibody screening strategies are not well suited to counteract the rapid mutation of SARS‐CoV‐2. Therefore, there is an urgent need to screen better broad‐spectrum neutralizing antibody. In this study, a comprehensive approach to design broad‐spectrum inhibitors against both SARS‐CoV‐1 and SARS‐CoV‐2 by leveraging the structural diversity of nanobodies is proposed. This includes the de novo design of a fully human nanobody library and the camel immunization‐based nanobody library, both targeting conserved epitopes, as well as the development of multivalent nanobodies that bind nonoverlapping epitopes. The results show that trivale B11‐E8‐F3, three nanobodies joined tandemly in trivalent form, have the broadest spectrum and efficient neutralization activity, which spans from SARS‐CoV‐1 to SARS‐CoV‐2 variants. It is also demonstrated that B11‐E8‐F3 has a very prominent preventive and some therapeutic effect in animal models of three authentic viruses. Therefore, B11‐E8‐F3 has an outstanding advantage in preventing SARS‐CoV‐1/SARS‐CoV‐2 infections, especially in immunocompromised populations or elderly people with high‐risk comorbidities.

## Graphical abstract

This study presents the design and development of multivalent nanobodies targeting conserved epitopes of SARS‐CoV‐1 and SARS‐CoV‐2. The trivalent nanobody B11‐E8‐F3 exhibits broad‐spectrum neutralization against SARS‐CoV‐1 and multiple SARS‐CoV‐2 variants, including Omicron sublineages, and demonstrates significant preventive and therapeutic efficacy in animal models.

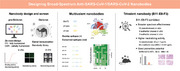

## 1 Introduction

From the first confirmed case of SARS in November 2002 to the end of the outbreak in August 2003, the World Health Organization (WHO) reported 8096 confirmed cases, with a 10% mortality rate.^[^ ^1^ ^]^ Sixteen years later, the highly similar severe acute respiratory syndrome coronavirus 2 (SARS‐CoV‐2) unexpectedly appeared in a more destructive manner and corona virus disease 2019 (COVID‐19) became a human “public health emergency of international concern.” As of 12 October 2023, the WHO had reported more than 770 million confirmed cases of COVID‐19, including approximately seven million deaths.^[^ ^2^ ^]^ SARS‐CoV‐2 is more contagious, and as most people worldwide have been infected, the virus has multiplied in hundreds of millions of ‘human petri dishes’. Under evolutionary pressure, it continues to evolve into new variants with enhanced infectivity and immune escape.^[^ ^3^ ^]^ The most noteworthy are Omicron spike (S) proteins have accumulated more than 30 amino acid mutations and have rapidly emerged in different evolutionary branches around the world, such as BF.7, BQ.1.1, and XBB.1.5,^[^ ^4^ ^]^ which are more infectious and immune evasive. Therefore, we cannot predict the future mutation direction and pathogenicity of SARS‐CoV‐2, which brings great challenges to the development of vaccines and drugs.

Since the outbreak of COVID‐19, various antibodies against SARS‐CoV‐2 have been approved for diagnosis and treatment.^[^ ^5^ ^]^ However, SARS‐CoV‐1/SARS‐CoV‐2 are both positive‐sense single‐stranded RNA viruses ((+) ssRNA) of the genus *Betacoronavirus* and are highly mutable.^[^ ^6^ ^]^ Once a mutation occurs in key areas of antibody recognition, such as the N501Y, Q493K, D614G, E484A, and S373P mutations, the neutralizing activity of the antibody drugs is reduced or even completely lost.^[^ ^7^ ^]^ Most neutralizing antibodies mainly recognize the receptor‐binding region (RBD) of the viral S protein, blocking the RBD from binding to human ACE2 receptors, preventing the virus from entering cells.^[^ ^8^ ^]^ Studies have also reported neutralizing antibodies that recognize the N‐terminal domain regions of the S protein^[^ ^9^ ^]^ but have been shown to lose neutralizing activity against the Alpha, Beta, and Delta variants.^[^ ^10^ ^]^ In addition to the neutralizing antibodies reported in the literature as ineffective against multiple variants,^[^ ^11^ ^]^ antibody drugs conditionally marketed through green channels also face great challenges. Casirivimab/imdevimab (Roche and Regeneron Pharmaceuticals) and bamlanvimab/etesevimab (Eli Lilly Pharmaceutical), two cocktail antibody drugs, and sotrovimab (Vir Biotechnology) have been granted emergency use authorization from the United States Food and Drug Administration (FDA) but was soon withdrawn for emergency use due to large variability of Omicron resulting antibodies inactivation.^[^ ^12^ ^]^ Given the unique advantages of neutralizing antibodies, it remains crucial to identify candidates with broad‐spectrum neutralizing effects.First, unlike vaccines, which are primarily preventive, neutralizing antibodies can both treat infected patients and provide passive immunity to susceptible individuals. Second, neutralizing antibodies are immediately effective after receiving an injection, especially in patients who have a weak or no response to the vaccine. Lastly, antibody drugs have high specificity and safety and have achieved positive results against many infectious diseases in clinical applications.^[^ ^13^ ^]^

During the boom in development of SARS‐CoV‐2 antibody drugs, nanobodies attracted much attention because they have unique characteristics compared to traditional antibodies, such as a smaller size, stronger penetration, lower immunogenicity, and longer complementarity‐determining region 3 (CDR3) length, making it possible for them to reach epitopes which were not accessible to conventional antibodies.^[^ ^14^ ^]^ Moreover, it is easy to design bivalent or multivalent nanobodies against the same or different antigen epitopes, particularly polymeric proteins, thereby producing powerful and broad‐spectrum antiviral drugs.^[^ ^14b^ ^]^ Therefore, screening broad‐spectrum nanobodies against SARS‐CoV‐1/SARS‐CoV‐2 is of great significance.

This study proposed the screening of broad‐spectrum anti‐SARS‐CoV‐1/SARS‐CoV‐2 multivalent nanobodies. First, we obtained three broad‐spectrum anti‐SARS‐CoV‐1/SARS‐CoV‐2 nanobody candidates (i.e., SN‐F3, ZF‐B11, and ZF‐E8) based on de novo design and the camel immunization nanobody library and evaluated their functional properties against SARS‐CoV‐1/SARS‐CoV‐2 pseudoviruses and authentic viruses. Second, a series of multivalent nanobodies (bivalent and trivalent) were designed based on the three nanobody candidates. It was finally determined that B11‐E8‐F3, the three nanobodies concatenated in tandem into a trivalent form, had the broadest spectrum and efficient neutralization activity. The pseudovirus neutralization spectrum spans from SARS‐CoV‐1 to 14 SARS‐CoV‐2 variants of concern (VOCs), i.e., SARS‐CoV‐2 (WT), Alpha, Beta, Gamma, Delta, Omicron sublineages (and even for the recently globally circulating XBB.1.16 and EG.5 strains) are also fully covered. Moreover, B11‐E8‐F3 exhibits broader protection against authentic viruses, including six authentic viruses in vitro and three authentic viruses in vivo. Therefore, B11‐E8‐F3 holds promise as a next‐generation broad‐spectrum antiviral drug against both SARS‐CoV‐1 and SARS‐CoV‐2.

## 2 Results

### 2.1 De Novo Design, Construction, and Screening of a Broad‐Spectrum Anti‐SARS‐CoV‐1/SARS‐CoV‐2 Nanobody Synthesis Library

**Figure 1:**
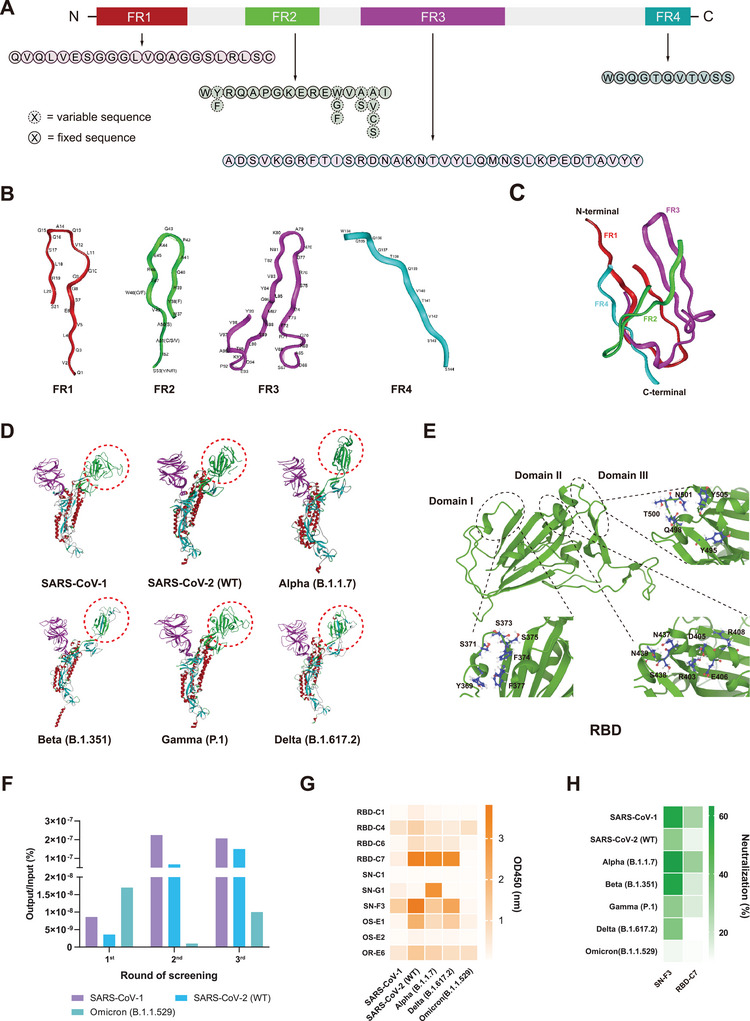
De novo design of fully human nanobody synthetic library and screening of broad‐spectrum nanobody candidates. A) Sequences of generalized human nanobody FRs (FR1, FR2, FR3, and FR4). B) The 3D theoretical structures of generalized human nanobody FRs (FR1, FR2, FR3, and FR4) using computer‐aided homology. C) The 3D combinational structure and assigned orientation of the chosen FRs were constructed using structural superimposition. D) The theoretical complex structures of six S proteins optimized by CVFF force field using Discover_3. RBDs are marked by red dotted circle. The crystal structures were chosen as follows: SARS‐CoV‐1 PDB code,7LM9; SARS‐CoV‐2(WT) PDB code, 6VXX; Alpha PDB code, 7DX1; Beta PDB code, 7WEV; Gamma PDB code, 8DLO; Delta PDB code, 7W92. E) Three conserved structural domains on RBD of six S proteins, i.e., Domain I: Y369, S371, S373, F374, S375, and F377; Domain II: R403, D405, E406, R408, N437, S438, and N439; Domain III: Y495, Q498, T500, N501, and Y505. F) Output/Input ratio of three rounds phage screening to enrich positive clones based on RBD proteins (SARS‐CoV‐1/SARS‐CoV‐2(WT)/Omicron). G) Binding activity of 10 human nanobody candidates (fused with Fc) to five RBD/S proteins of SARS‐CoV‐1/SARS‐CoV‐2 variants evaluated by ELISA (*n* = 2). (H) Neutralizing activity of two human nanobody candidates against seven HIV pseudoviruses of SARS‐CoV‐1/SARS‐CoV‐2 variants (*n* = 3).

A general synthesis nanobody library against SARS‐CoV‐1/SARS‐CoV‐2 was constructed theoretically by de novo design. First, 1185 nanobody sequences were collected and classified using multiple sequence alignment analysis. As shown in Figure S1A, Supporting Information, the amino acids in the framework region (FR) are relatively conserved and highly homologous with the human heavy‐chain antibody variable region HV3. Subsequently, we determined the suitable human nanobody FR by considering the nanobody’s fully human FR characteristics and the classification principles of Kabat and IMGT. As shown in **Figures** **1**A and S1A (Supporting Information), the hydrophilic amino acids E and R in the sequence from camel was retained in FR2, while F was changed to W, and C was introduced to form an additional disulfide bond with the potential C in CDR3, and some amino acids in FR3 and FR4, which did not affect the framework structure but had more amino acids than VH3, were deleted to make it more similar to the human antibody framework. Furthermore, as shown in Figure 1B, to ensure the correct structural connection and suitable conformational match between the FRs and CDRs, the 3D theoretical structures of the FR were modelled using a computer‐aided homology method. Based on the 3D crystal structures of the nanobodies deposited in the Protein Data Bank (PDB), a 3D combinational structure and assigned orientation of the chosen FRs were constructed using structural superimposition, as shown in Figure 1C. The S protein structures of SARS‐CoV‐1 and SARS‐CoV‐2 pre‐Omicron variants were obtained from the PDB database and optimized with the Discover_3 program under CVFF force field and shown in Figure 1D. Using the IEDB website (https://iedb.org), three potentially important RBD (pre‐Omicron strains) domains with relatively conserved sequences and structures that interact with ACE2 were determined and shown in Figure 1E, Domain I spans from V362 to C369 at the N‐terminus of the RBD, where Y369, S371, S373, F374, S375, and F377 are relatively conserved and externally exposed amino acids. Domain II was identified as an amino acid sequence from R403 to D442 at the middle of the RBD, where R403, D405, E406, R408, N437, S438, and N439 are relatively conserved, externally exposed amino acids. Domain III was identified as an amino acid sequence from L492 to Y508 at the C‐terminus of the RBD, where Y495, Q498, T500, N501, and Y505 are relatively conserved, externally exposed amino acids. Then, CDRs were designed based on the physicochemical properties of the residues at the corresponding positions of ACE2 and the aforementioned key domains (I, II, and III) of the RBD and their interacting structural features. In detail, to enhance the intermolecular hydrogen bonds between potential CDR1 and domain I, polar residues such as Ser, Asn, and Tyr were introduced into CDR1, and similar charged and aromatic residues were mutated.Since domain II contains charged residues such as Asp, Arg, and His, these were introduced into the predicted CDR2 library. In addition, considering the CDR3 properties of the nanobody, polar and charged. We also determined the amino acid lengths for CDR1, CDR2, and CDR3 to be 14, 11, and 34, respectively. Residues were introduced and the length was extended to enhance the intermolecular interaction. Furthermore, domains II and III were considered as targets to design the CDR3. According to the complex crystal structure of ACE2 and SARS‐CoV‐2 spike protein (PDB code: 6LZG), the important amino acids in ACE2 involved in binding to the RBD epitope were assigned the key position of the CDRs, and the positively charged residues (i.e., Lys and Arg), negatively charged residues (i.e., Asp and Glu) and the aromatic residues (i.e., Phe and Tyr) were the main constituents. Dealing with the rule, the adjacent positions were placed with similar physicochemical property residues as potential mutants, and some positions that near the FRs were used as potential fixed residues. We also determined the amino acid lengths for CDR1, CDR2, and CDR3 to be 14, 11, and 34, respectively. Notably, the CDR3 was set to 34 amino acids in order to allow the formation of a larger stem‐loop structure and thus a larger area of action with the antigen, which is less prone to escape due to mutation of the virus. A fully human nanobody library with SARS‐CoV‐1/SARS‐CoV‐2 bias was finally obtained, as shown in Figure S1B. Subsequently, this library was constructed by designing degenerate primers and overlap‐PCR and electro‐transfection with library capacity of 8 × 10^10^. Then, 100 clones were selected for sequencing analysis, and 65 valid sequences were aligned as shown in Figure S1C (Supporting Information).The site of mutation, the type of amino acid, and the frequency of amino acid occurrence were consistent with the de novo design, indicating that the library has been successfully constructed. Then, three rounds of phage screening were performed, as the results shown in Figure 1F, the positive clones of SARS‐CoV‐1, SARS‐CoV‐2 (WT) were significantly enriched during the phage screening process, whereas there was no enrichment effect of the Omicron protein during several rounds of the screening process. Ten sets of nanobody sequences were finally obtained by sequencing the positive clones, and the corresponding nanobodies were expressed with Fc and verified, their binding activities with five S proteins of variants (SARS‐CoV‐1, SARS‐CoV‐2 (WT), Alpha, Beta, and Delta) (Figure 1G). SN‐F3 and RBD‐C7 exhibited good recognitions to more than three proteins. Subsequently, seven pseudoviruses based on human immunodeficiency virus (HIV) skeleton (SARS‐CoV‐1, SARS‐CoV‐2 (WT), Alpha, Beta, Delta, and Omicron) were used for evaluating neutralizing activity. The results showed (Figure 1H) that only SN‐F3 had a broader spectrum of neutralizing activity, except for weak neutralizing activity against Omicron (7/7). Therefore, SN‐F3 was selected for further studies.

### 2.2 Camel Immunization, Construction, and Screening of Broad‐Spectrum Anti‐SARS‐CoV‐1/SARS‐CoV‐2 Nanobodies

**Figure 2:**
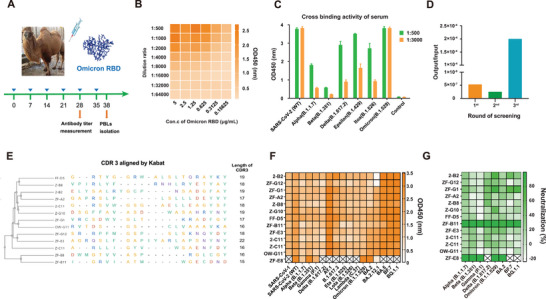
Construction of a camel immunized library and screening of broad‐spectrum nanobody candidates. A) Schematic diagram of camel immunization using Omicron RBD protein. B) Nanobody titers of immunized camel serum against Omicron RBD protein evaluated by ELISA. (C) Cross‐binding activity to S or RBD proteins of SARS‐CoV‐2 variants prior to 5th immunization evaluated by ELISA. D) Output/input ratio of three rounds phage screening to enrich positive clones for the Omicron RBD protein. E) Kabat alignment of CDR3 sequences of 12 nanobody candidates with potential binding/neutralizing activity. F) Binding activity of 12 nanobody candidates to S/RBD proteins of SARS‐CoV‐1 and 16 SARS‐CoV‐2 variants by ELISA. The symbol “×” indicates no binding activity. G) Neutralizing activity of 12 nanobody candidates against eight HIV pseudoviruses of SARS‐CoV‐2 variants (*n* = 3). The symbol ‘×’ indicates no neutralizing activity.

Considering the increased immune escape ability of Omicron sublineages, the Omicron RBD protein was used to immunize camels. After the fifth immunization, we collected serum samples and measured the titer. The results showed that camels produced high antibody titers against the immunized antigens (**Figure** **2**A,B), which exhibited high cross‐binding activity against multiple RBD proteins of variants (Figure 2C), indicating higher abundance of nanobodies specifically directed against SARS‐CoV‐2. After three rounds of phage screening (Figure 2D), 163 positive clones with different sequences were obtained. Subsequently, 50 representative sequences were selected according to the similarity of CDR3 sequences. The evolutionary tree and the corresponding CDR3 of each sequence are shown in Figure S2A (Supporting Information). Subsequently, 50 nanobodies were expressed with Fc and verified for binding activity. Figure S2B (Supporting Information) shows that most of the candidates can efficiently bind to six RBD proteins (Delta, Omicron, BA.2, BA.2.12.1, BA.4, and BA.5). Furthermore, Beta and BA.2 of HIV pseudoviruses were selected for initial neutralization activity verification (Figure S2C, Supporting Information), 13 nanobodies were selected that exhibited neutralizing activity against at least one of the pseudoviruses as mentioned previously; the corresponding code and amino acid sequence of the CDR3s are shown in Figure 2E. After obtaining the purified nanobodies, their binding and neutralizing activities were further determined, and the results (Figure 2F,G) showed that ZF‐B11 almost completely covered protein binding and pseudovirus neutralization (17/17, 8/8), whereas ZF‐E8 only partially effective (7/17, 5/8). Although ZF‐E8 lost its binding activity to Delta and some Omicron sublineages. Nevertheless, ZF‐E8 had excellent neutralizing activity against other VOCs, even better than that of ZF‐B11. Therefore, ZF‐B11 and ZF‐E8 were selected for further studies.

### 2.3 SN‐F3, ZF‐B11, ZF‐E8 are Active Across SARS‐CoV‐1/SARS‐CoV‐2 In Vitro

**Figure 3:**
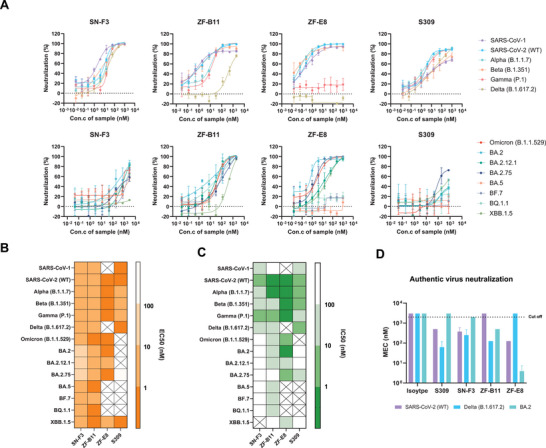
Neutralizing activity of SN‐F3, ZF‐B11, and ZF‐E8 against SARS‐CoV‐1 and SARS‐CoV‐2 variants in vitro. A,C) Neutralizing activity of SN‐F3, ZF‐B11, and ZF‐E8 against HIV pseudoviruses (*n* = 3). C) IC50 of neutralizing assay. The symbol ‘×’ represents no neutralizing activity. S309 is the control antibody approved by FDA for emergency clinical use. B) EC50 of SN‐F3, ZF‐B11 and ZF‐E8 binding to SARS‐CoV‐1 and 13 SARS‐CoV‐2 VOCs/VOIs evaluated by ELISA (*n* = 2). The symbol ‘×’ represents no binding activity. D) Neutralizing activity of antibody against three authentic viruses in Vero E6 cells evaluated by inhibition of cytopathic effect (CPE) (*n* = 3). The minimum effective concentration (MEC) was judged by the absence of significant CPE. The cutoff value represents the highest concentration tested that did not fully inhibit the CPE.

We further confirmed the in vitro biological activity of SN‐F3, ZF‐B11, ZF‐E8 selected by two different technological paths. In follow‐up studies, we mainly focused on VOCs published by the WHO. To better evaluate the activity of the nanobody candidates, S309, the aforementioned clinical emergency‐approved broad‐spectrum neutralizing antibody sotrovimab, was selected as a control.^[^ ^15^ ^]^ First, we verified the three nanobodies with the correct molecular mass and high protein purity through SDS‐PAGE and HPLC (Figures S3A,B, Supporting Information). Second, we evaluated the binding activity of the three nanobody candidates against 14 proteins (Figure S3C, Supporting Information), and the corresponding EC_50_ values are presented in Figure 3B and Table S2 (Supporting Information). S309 only retained binding activity against several pre‐Omicron VOCs and lost binding activity against most Omicron sublineages (8/14). In contrast, the three nanobody candidates exhibited a broader spectrum of binding activity. SN‐F3 and ZF‐B11 almost completely covered the protein binding tested (14/14), with most EC_50_ less than 10 × 10^−9^ m. Although ZF‐E8 only partially covered the protein binding tested (9/14), the EC_50_ to 7 proteins was less than 1 × 10^−9^ m.

In addition, 14 HIV pseudoviruses with SARS‐CoV‐1/SARS‐CoV‐2 S protein variants were prepared to measure the neutralizing activity. As shown in **Figure** **3**A,C, and Table S2 (Supporting Information), ZF‐B11 could effectively neutralized each type of pseudoviruses tested (14/14), whereas SN‐F3 and ZF‐E8 neutralized less of pseudoviruses tested (13/14, 9/14). SN‐F3 had a strong neutralizing effect on the pre‐Omicron mutant strains, with IC_50_ less than 25 × 10^−9^ m, while only showing weak neutralizing activity against Omicron at high concentrations (IC_50_>100 × 10^−9^ m). In contrast, ZF‐B11 had stronger neutralizing activity against 11 variants with IC_50_ lower than 100 × 10^−9^ m, even for five of the variants with IC50 values below 25 × 10^−9^ m. Although ZF‐E8 did not have broad‐spectrum neutralizing activity, the IC_50_ values for the 7 pseudoviruses measured were lower than 4 × 10^−9^ m, whereas S309 almost lost neutralizing activity against Omicron sublineages, with most of the IC50>500 × 10^−9^ m, and considered not to have neutralizing activity. In addition, although SN‐F3 could bind to most of the Omicron viruses, its counterpart has low or no neutralizing activity against them. To more intuitively determine the neutralizing activity of the three candidates, we constructed a green fluorescent pseudovirus system based on the VSV skeleton, mainly including SARS‐CoV‐2 (WT), Delta, and Omicron. Similar results are shown in Figure S3D (Supporting Information).

Furthermore, we evaluated the authentic viral (SARS‐CoV‐2 (WT), Delta, and BA.2) neutralizing activities of the three nanobody candidates. Figure S3E (Supporting Information) shows the pictures of the viruses producing a cytopathic effect (CPE) under specific concentration of antibody, and the numbers on the graph indicate that the cells start to produce CPE at that concentration. Furthermore, Figure 3D shows the minimum effective concentration (MEC) of each antibody (i.e., the concentration which does not produce a CPE). It can be seen from the results that SN‐F3 was effective against three viruses, and ZF‐B11 and ZF‐E8 were effective against two viruses. The MEC of three nanobodies against the WT, Delta, and BA.2 viruses were SN‐F3 (375 ± 216.51 × 10^−9^, 250 ± 216.51 × 10^−9^, 2000 ± 0 × 10^−9^ m), ZF‐B11 (∞, 125 ± 0 × 10^−9^, 500 ± 0 × 10^−9^ m), and ZF‐E8 (125 ± 0 × 10^−9^ m, ∞, 3.90 ± 3.38 × 10^−9^ m), respectively. Thus, the three nanobodies have the advantage of complementary neutralization against the three viruses and the results are presented in Table S3 (Supporting Information).

### 2.4 In Vivo Characterization of Nanobody Candidates

To evaluate the neutralizing activity of the three nanobodies in vivo, we established a VSV‐based luciferase reporter pseudoviral infection hamster model. A replication‐competent reporter virus was used to intranasally infect hamsters. Hamsters were intraperitoneally injected with S309, SN‐F3, ZF‐B11, ZF‐E8, or isotype control antibodies at a dose of 10 mg/kg 24 h before intranasal infection with VSV‐pseudoviruses. Hamster blood was collected at 24 or 12 h intervals for the detection of nanoluciferase activity. At the viral replication peak time confirmed by pre‐experiment, the hamsters were subjected to Living Image Software (IVIS) spectrum scanning. As shown in Figure S4A–C (Supporting Information), S309, SN‐F3, ZF‐B11, and ZF‐E8 significantly inhibited VSV‐∆G‐SARS‐CoV‐2 S (WT)‐NanoLuc viral infection compared to that of the isotype control. The luminescence intensity was also quantified using IVIS software. Figure S4B (Supporting Information) shows that, the four antibodies tested had potent inhibitory effects on VSV‐∆G‐SARS‐CoV‐2 S (WT)‐NanoLuc viral infection. Consistent with the IVIS image, SN‐F3, ZF‐B11, and ZF‐E8 had better inhibitory effects than S309 on nanoluciferase activity assay (Figure S4C, Supporting Information). The blood nanoluciferase activity revealed that the virus replicated to the peak level at 24 h postinfection, begins to fall back at 48 h post‐infection and drops to background values 72 h postinfection. Similar experiments were performed on Delta and Omicron variants. ZF‐E8 was not tested on Delta, and Figure S4D (Supporting Information) shows that S309, SN‐F3, and ZF‐B11 blocked VSV‐∆G‐SARS‐CoV‐2 S (Delta)‐NanoLuc viral infection compared to the isotype control. Furthermore, the luminescence intensity and hamster blood nanoluciferase activity revealed that SN‐F3 and ZF‐B11 had more potent inhibitory effects than S309 (Figures S4E,F, Supporting Information). For the Omicron pseudovirus, SN‐F3 and S309 almost completely lost their protective effects. However, ZF‐B11 and ZF‐E8 which originated from the Omicron RBD protein‐immunized camels, still had inhibitory effects, as shown in Figure S4G–I (Supporting Information). Collectively, these three nanobody candidates have potent inhibitory effects on VSV‐based pseudoviral infections.

### 2.5 Epitope Analysis of Nanobody Candidates

**Figure 4:**
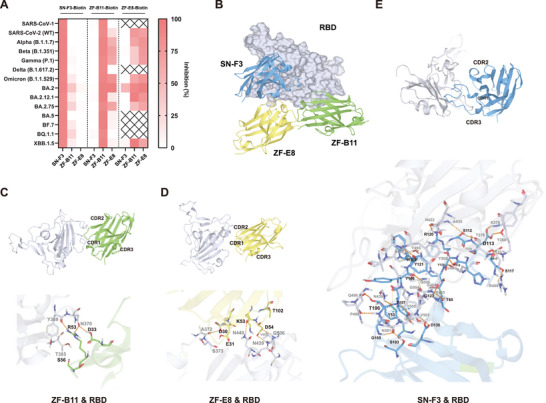
Epitope characteristics of SN‐F3, ZF‐B11, and ZF‐E8. A) Epitope overlap between SN‐F3, ZF‐B11, and ZF‐E8 on the S/RBD proteins of SARS‐CoV‐1/SARS‐CoV‐2 VOCs/VOIs evaluated by competitive ELISA (*n* = 2). The symbol ‘×’ indicated biotinylated antibodies with weak (<20%) or no binding activity. B) Structure model of three nanobodies binding to RBDSARS‐CoV‐2 (WT) protein. The RBD protein is depicted as gray surface. Nanobodies are shown as ribbons (blue: SN‐F3, green: ZF‐B11, yellow: ZF‐E8). C–F) Interaction between three nanobodies and RBD protein of SARS‐CoV‐2 (WT). RBD proteins are shown as gray ribbons. The residues involved in interactions are represented as sticks. Interaction forces are shown as dotted line (orange: hydrogen bonds, yellow: weak hydrogen bonds, blue: pi‐pi stacking).

Based on the results presented above, it could be concluded that the three selected nanobodies have broad‐spectrum antiviral activity with different neutralization profiles. Subsequently, we investigated whether there was any epitope overlap among these nanobodies and their recognition characteristics with the RBD. First, we tested whether each of the three nanobodies have epitope crossover (**Figure** **4**A). ZF‐B11 and ZF‐E8 showed a tendency to compete on most VOCs, whereas SN‐F3 showed nearly no competition with the two nanobodies. Second, we computationally simulated the antigen–nanobody interactions of three complexes, namely ZF‐B11 and RBD, ZF‐E8 and RBD, and SN‐F3 and RBD, and conducted an in‐depth analysis of their interaction patterns. Figure 4B illustrates the recognition pattern between the three nanobodies and RBD. From the results, it is evidently that the core epitopes recognized by these nanobodies do not significantly overlap. However, this contradicts our competition experiments where ZF‐B11 and ZF‐E8 compete with each other. Therefore, we further analyzed the key amino acid residues involved in each individual complex's interaction. Figures 4C–E demonstrates the interface amino acid residues involved in antigen‐nanobody interactions. Among them, ZF‐B11 and ZF‐E8 mainly interact with CDR1 and CDR2 regions of RBD while SN‐F3 predominantly interacts with CDR3 region of RBD; moreover, SN‐F3 exhibits more interaction sites than others. Table S4 (Supporting Information) summarizes both the interacting amino acids between antigens and nanobodies as well as their corresponding interaction forces. ZF‐B11 recognizes Domain I of RBD whereas both ZF‐E8 and SN‐F3 recognize Domain I/II/III regions of RBD. Additionally, it was observed that ZF‐B11 recognizes Y369 and N370 on Domian I while ZF‐E8 similarly recognizes A372 and S373 on Domian I. The regions on Domain I recognized by both nanobodies are spatial proximity, resulting in certain spatial hindrance which provides an explanation for their competitive behavior observed in previous experiments.

### 2.6 Multivalent Nanobodies Exhibit Broader Spectrum and More Efficient Neutralization Activity In Vitro

**Figure 5:**
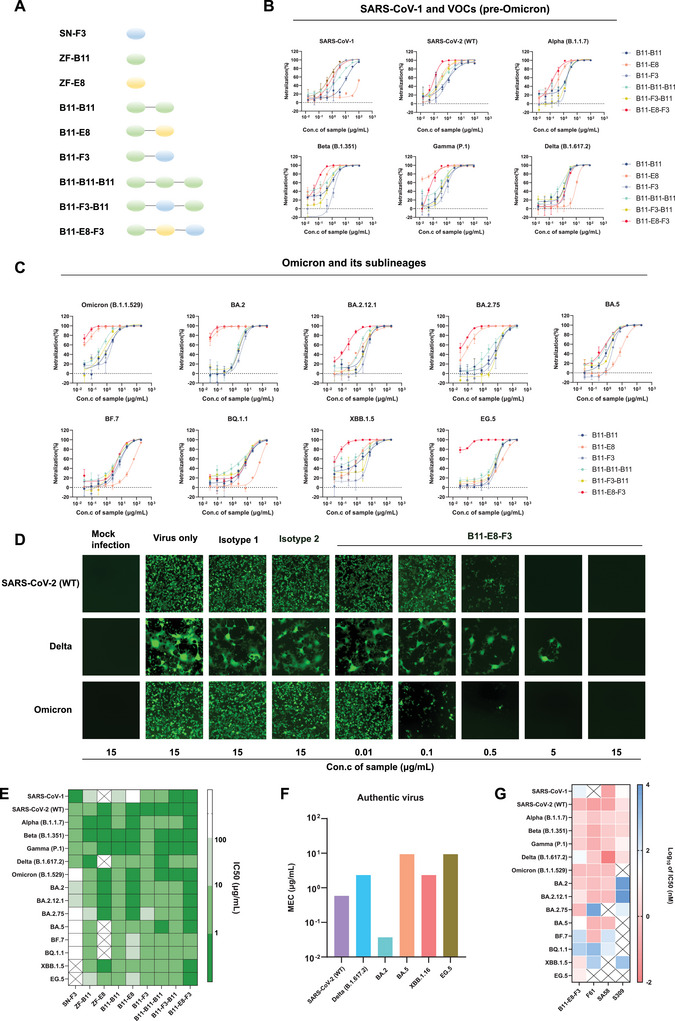
Neutralizing activity of multivalent nanobodies against pseudoviruses and authentic viruses. A) Schematic diagram of mutivalent nanobodies. The oval shape with different colours represents monovalent subunits. Blue, SN‐F3; Green, ZF‐B11; Yellow, ZF‐E8. (G4S)n link is showed with a gray line, bivalent nanobodies are linked by (G4S)2, and trivalent nanobodies are linked by (G4S)3. B,C, and E) Neutralizing activity of multivalent nanobodies against HIV pseudoviruses of SARS‐CoV‐1 and 14 SARS‐CoV‐2 VOCs/VOIs as described previously (*n* = 3). B) Neutralizing activity of SARS‐CoV‐1 and five pre‐Omicron VOCs pseudovirus. C) Neutralizing activity of nine Omicron VOCs/VOIs pseudovirus. E) IC50 of neutralizing assay. The symbol ‘×’ indicates no neutralizing activity. D) Neutralizing activity of B11‐E8‐F3 against three VSV‐GFP pseudoviruses (WT/Delta/Omiron) evaluated by intensity and distribution of GFP in Vero E6 cells (200×). Isotope 1 is a clinically investigational anti‐ricin antibody drug developed in our laboratory. Isotype 2 is a commercialized anti‐HER2 monoclonal antibody drug (Herceptin). F) The MEC of B11‐E8‐F3 inhibiting 6 authentic viruses entering into Vero E6 cells as described previously (*n* = 3). G) IC50 of neutralisation assay of three reported broad‐spectrum neutralizing antibodies and B11‐E8‐F3 against 15 HIV pseudoviruses (*n* = 3). The symbol ‘×’ indicates no neutralizing activity.

Considering that SN‐F3 does not overlap with the recognition epitopes of ZF‐B11 and ZF‐E8, and that the three nanobodies have different neutralizing viral profiles, multivalent nanobody design could be considered. We obtained bivalent (≈30 kDa) or trivalent (≈45 kDa) nanobodies (**Figure** **5**A). In addition to the 14 HIV pseudoviruses mentioned before, we established EG.5 pseudoviruses, as new variants of Omicron continue to be reported. As shown in Figure 5B,C and Figure S6A (Supporting Information), multivalent nanobodies had stronger neutralizing activity and wider virus coverage than monovalent nanobodies. Further, Figure 5E demonstrates a heat map of the IC_50_ values of the neutralizing activity, and the results show that all the multivalent nanobodies provide full coverage for tested VOCs/VOIs pseudoviruses (15/15) when compared to the monovalent nanobodies. It is noteworthy that the tandem connection of three nanobodies to form the trivalent nanobody B11‐E8‐F3 has significantly improved broad‐spectrum and neutralization activity. Moreover, the IC_50_ of B11‐E8‐F3 for 12 pseudoviruses was lower than 1 µg mL^−1^. Clearly, B11‐E8‐F3 had the recognition characteristics of the three nanobodies, theoretically covering a wider range of viral spectra, and further improving the neutralizing activity against most pseudoviruses compared to monovalent or bivalent nanobodies. Moreover, it seems that the introduction of SN‐F3 in B11‐E8‐F3 also enhances its neutralization activity to some extent (e.g., EG.5) compared to B11‐E8, despite the fact that SN‐F3 has lower neutralization activity for the vast majority of Omicrons, possibly because the trivalent antibody increases spatial site‐blocking in the recognition space, which in turn inhibits the ACE2/S protein interactions more efficiently. Further, we used the aforementioned VSV‐ΔG‐SARS‐CoV‐2 S‐GFP (VSV‐GFP) pseudoviruses to visualize the neutralizing activity. As shown in Figure S6B–D (Supporting Information), the neutralizing activity of multivalent nanobodies was significantly better than that of monovalent antibodies. Moreover, B11‐E8‐F3 had the strongest neutralizing effect and showed notable viral inhibition when the antibody concentration ranged from 0.1 to 0.5 µg mL^−1^ (Figure 5D). We also tested the neutralizing activity of B11‐E8‐F3 against six authentic viruses, SARS‐CoV‐2 (WT), Delta, BA.2, BA.5, XBB.1.16, and EG.5 (Figure 5F). B11–E8–F3 showed good neutralization activity against all six authentic viruses, especially against BA.2 with MEC values as low as 0.037 µg mL^−1^. Next, we determined that the affinity of B11‐E8‐F3 for the above mentioned 15 RBD proteins was at the nanomolar level and confirmed that B11‐E8‐F3 exerts its efficacy by blocking ACE2/RBD protein interactions (Figure S5A,B, Supporting Information). Further, in order to better demonstrate the broad‐spectrum antiviral activity of the B11‐E8‐F3, we compared B11‐E8‐F3 with three reported broad‐spectrum neutralizing antibodies, F61,^[^ ^16^ ^]^ SA58,^[^ ^17^ ^]^ and S309, against the HIV pseudoviruses. The IC_50_ values are shown in Figure 5G and Table S5 (Supporting Information). B11‐E8‐F3 achieved full coverage of the pseudoviruses measured (15/15), F61 had full coverage of SARS‐CoV‐2 pseudoviruses except for SARS‐CoV‐1 and EG.5 (13/15), SA58 was inactive against three Omicron pseudoviruses (12/15), and S309 was inactive for the vast majority of Omicron pseudoviruses (10/15). Overall, trivalent nanobodies B11‐E8‐F3 have broader spectrum SARS‐CoV‐1/SARS‐CoV‐2 neutralizing activity.

### 2.7 Preventive Activity of Multivalent Nanobodies Against Authentic Viruses

**Figure 6:**
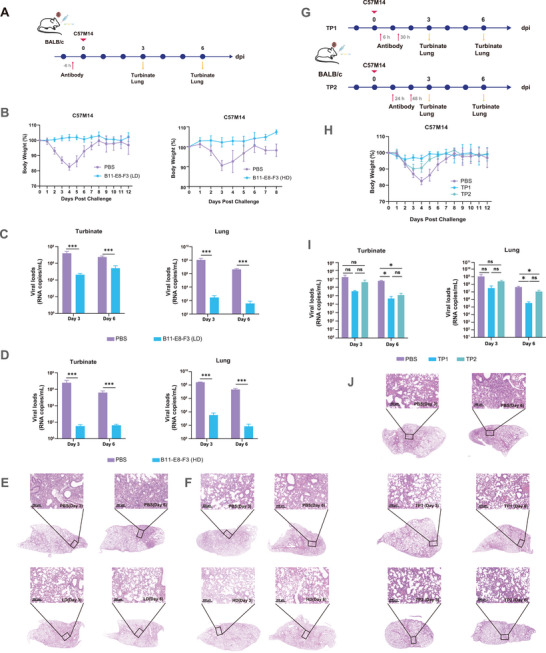
Preventive and therapeutic activity of B11‐E8‐F3 against SARS‐CoV‐2(WT) adapted authentic virus strain C57M14 in BALB/c mice. A–F) Preventive activity of B11‐E8‐F3 against C57M14 in BALB/c mice (*n* = 10). C57M14 is a mouse‐adapted strain of SARS‐CoV‐2 (WT) obtained through serial passaging in mice. A) Preventive experimental procedures. Mice were intranasally administered with a low‐dose (LD, 50 µg per mouse) or high dose (HD, 280 µg per mouse) of B11‐E8‐F3 6 h before intranasal virus challenge (10^4^ TCID50). Turbinates and lungs were harvested on days 3 and 6 for testing. B) Body weight of mice. C,D) Viral loads in turbinate and lungs of mice determined by qRT‐PCR (*n* = 3). E,F) HE staining of lungs. Scale bar, 200 µm. Statistical significance was analyzed using two‐sided unpaired Student's *t* tests. ***p < 0.001. G–J) Therapeutic activity of B11‐E8‐F3 against C57M14 in BALB/c mice (*n* = 10). G) Therapeutic experimental procedures. Mice in group TP1 were intranasally administered with B11‐E8‐F3 (280 µg/per mouse) at 6 and 30 h after virus challenge (10^4^ TCID50), and mice in group TP2 were treated intranasally with B11‐E8‐F3 at 24 and 48 h after virus challenge (10^4^ TCID50). Turbinates and lungs were harvested on days 3 and 6 for testing. H) Body weight of mice. I) Viral loads in turbinate and lung of mice determined by qRT‐PCR (*n* = 3). J) HE staining of lungs. Scale bar, 200 µm. Statistical significance was analysed using two‐sided unpaired Student's *t* tests. ns, no significance; *p < 0.05; **p < 0.01; ***p < 0.001.

It is clear that B11‐E8‐F3 has very broad‐spectrum and efficient antiviral activity from the above results. Next, we would like to determine whether B11‐E8‐F3 had more potential as prevention or treatment. We established a C57M14 authentic virus (i.e., SARS‐CoV‐2, WT) BALB/c mouse infection model to evaluate the preventive effect of B11‐E8‐F3 nasal administration 6 h in advance. A low dose (LD; 50 µg per mouse) and a high dose (HD; 280 µg per mouse) were used, and the results are shown in Figure S6A‒F (Supporting Information). The body weight in the PBS group decreased from the first day of inoculation to the maximum on the third or fourth day, with a weight loss of ≈10–15%, whereas both LD and HD groups showed some increase in body weight (**Figure** **6**B). Further observation of the turbinate and lung viral load on days three and six after infection showed that their viral loads were close to background values, except for the LD group, where small amounts of virus were detectable in the turbinate (Figure 6C,D), whereas the viral RNA copies in the PBS group reached 10^7^ ‒ 10^8^ (*p* < 0.001). Further, we conducted a histopathological examination of the lungs on the third and sixth days post‐infection (Figure 6E,F). Both prevention groups showed significantly less inflammation compared to PBS group. By the sixth day, specially, the LD group exhibited marked improvement in inflammatory cell infiltration and hemorrhage, while the HD group maintained a relatively normal lung tissue structure.

**Figure 7:**
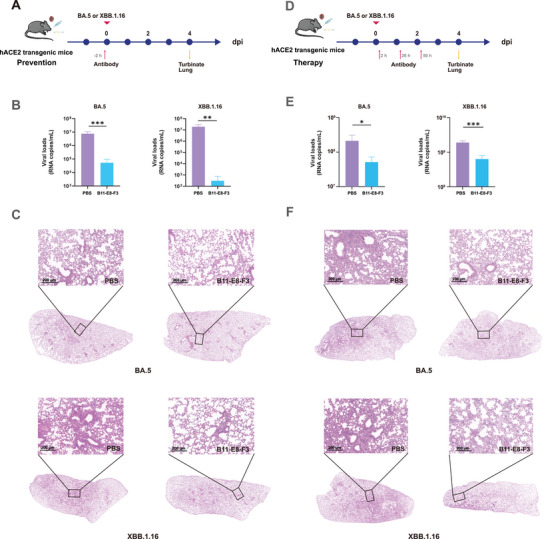
Preventive and therapeutic activity of B11‐E8‐F3 against BA.5 and XBB.1.16 authentic viruses in hACE2 transgenic C57BL/6J mice. A–C) Preventive activity of B11‐E8‐F3 against BA.5 (10^3^ TCID50) or XBB.1.16 (10^1.75^ TCID50) in hACE2 transgenic C57BL/6J mice (*n* = 4). Mice were intranasally administered with B11‐E8‐F3 2 h before the intranasal virus challenge. Lungs were harvested on day 4 for testing. A) Preventive experimental procedures. B) Viral load in lungs of mice determined by qRT‐PCR (*n* = 4). C) HE staining of lungs. Scale bar, 200 µm. Statistical significance for BA.5 was analyzed using two‐sided unpaired Student's *t* tests, and for XBB.1.16 using the Mann–Whiteny test (with unequal variances). *p < 0.05; **p < 0.01; ***p < 0.001. D–F) Therapeutic activity of B11‐E8‐F3 against BA.5 (10^4^ TCID50) or XBB.1.16 (10^2.75^ TCID50) in hACE2 transgenic C57BL/6J mice (*n* = 4). Mice were intranasally administered with B11‐E8‐F3 2 h, 26 h, and 50 h after the intranasal virus challenge. Lungs were harvested on day 4 for testing. D) Therapeutic experimental procedures. E) Viral load in the lungs determined by qRT‐PCR (*n* = 4). C) HE staining of lungs. Scale bar, 200 µm. Statistical significance was analyzed with two‐sided unpaired Student's *t* tests. *p < 0.05; **p < 0.01; ***p < 0.001.

We also carried out prevention experiments in human ACE2 (hACE2) transgenic mice (**Figure** **7**A–C). Compared with the PBS group, the lung viral load of mice receiving B11‐E8‐F3 prevention was reduced by 100‐and 10,000‐fold for BA.5 (***P < 0.001) and XBB.1.16 (**P = 0.009) infections, respectively (Figure 7B). The lung tissue of the PBS control group was infiltrated by large‐scale inflammatory cells, and the alveolar structure was destroyed, while only a small number of alveoli in the treatment group showed pathological damage accompanied by slight hemorrhage (Figure 7C), illustrating the intranasal administration of the B11‐E8‐F3 can effectively inhibit the replication of the virus in the lungs. These data indicates that B11‐E8‐F3 has a good preventive effect.

### 2.8 Therapeutic Capacity of Multivalent Nanobodies Against Authentic Viruses

Further, we assessed the therapeutic effects of B11‐E8‐F3. After C57M14 nasal infection, B11‐E8‐F3 were administered via nasal drops at 6/30 h (therapeutic group 1; TP1) and 24/48 h (therapeutic group 2; TP2) and the effect of the treatments were observed at different times (Figure 6G). The weight in the PBS group decreased to a minimum on the third or fourth day, with a weight loss rate of ≈20%, and recovered on day 8. In TP2, the weight loss also peaked on day three or four, reaching about 10%, and returned to normal after day 6. In contrast, mice in TP1 experienced only slight weight loss (less than 5%) between days one and four, and recovered by day five (Figure 6H). In addition, the viral load was suppressed more effectively in TP1 than in TP2 (Figure 6I). In the TP1, the viral load in the turbinates and lungs on the third day was nearly 100 times different from that in the PBS group, despite the lack of statistical significance (*p* = 0.125, *p* = 0.207), and the difference was greater on day six (*p = 0.027, *P = 0.029). In contrast, the viral load in the turbinates and lungs of TP2 were slightly reduced on the third day (*p* = 0.234 and *p* = 0.241, respectively), and the difference was greater than that of the PBS group on the sixth day (*P = 0.027 and *P = 0.017, respectively). The histopathological examination of the lungs showed significant improvement in inflammation in the TP1 and TP2 groups on day 3 and day 6 compared to the PBS group (Figure 6J). Overall, mice in TP1 recovered faster and had lower viral loads than those in TP2, indicating that B11‐E8‐F3 is effective against SARS‐CoV‐2 (WT), with earlier treatment providing better outcomes.

Subsequently, we continued to test B11‐E8‐F3 therapeutic effects against BA.5 and XBB.1.16 authentic viruses in hACE2 transgenic mice (Figure 7D). Following BA.5 or XBB.1.16 nasal infection, B11‐E8‐F3 was administered via nasal drops at 2, 26, and 50 hours to assess its therapeutic effects. As shown in Figure 7E, nasal injection could effectively inhibit the proliferation of BA.5 and XBB.1.16 viruses in the lungs (*P = 0.016, ***P < 0.001). Furthermore, lung pathology showed that the vehicle group of mice infected with BA.5 and XBB.1.16 also exhibited severe pathological damage in the lungs (Figure 7F), including thickening of the alveolar septa, inflammatory cell infiltration, and hemorrhage. After three consecutive days of B11‐E8‐F3 administration, the extent of the lesions was significantly reduced, with most of them concentrated around the blood vessels, indicating the resolution of inflammation. However, the suppression of viral load was not an exponential reduction, but only 2–3 times. A possible reason for this was that the viral titer of the challenge was too high, resulting in viral RNA copies in the PBS group up to 10^9^ mL^−1^. Therefore, the results indicate that B11‐E8‐F3 has good therapeutic effect in vivo.

### 2.9 Multivalent Nanobodies Exert Neutralizing Activity by Competing for ACE2/RBD

**Figure 8:**
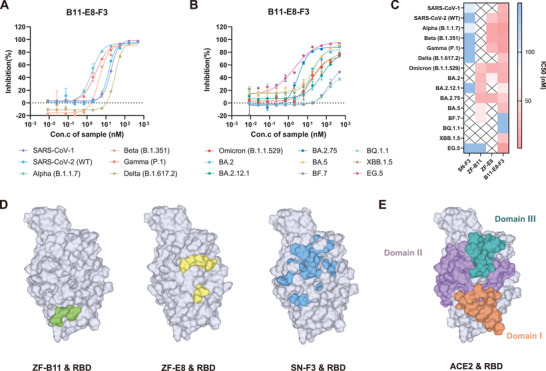
Mechanism of B11‐E8‐F3 neutralizing SARS‐CoV‐1/SARS‐CoV‐2. A,B) Competition between B11‐E8‐F3 with ACE2‐biotin on 15 RBD proteins (*n* = 2). C) IC50 of competition assay between nanobodies with ACE2‐biotin on 15 RBD proteins. The symbol ‘×’ indicates nanobodies had no competition with ACE2 (*n* = 2). D) Epitopes of three nanobodies with SARS‐CoV‐2(WT) RBD protein. Amino acid residues recognized by each nanobody was marked with different color (blue: SN‐F3, green: ZF‐B11, yellow: ZF‐E8). E) The core epitopes of ACE2 interacting with RBDSARS‐CoV‐2(WT) protein (orange: Domain I, purple: Domain II, turquoise: Domain III).

We further investigated whether B11‐E8‐F3 effectively blocked ACE2/RBD protein interactions, which is the key mechanism for antibodies to exert neutralizing effects. **Figures** **8**A,B demonstrate that B11‐E8‐F3 efficiently blocked the binding of 15 RBD proteins to ACE2. However, none of the individual nanobodies (SN‐F3, ZF‐E8, ZF‐B11) could completely compete with ACE2 for binding to both BQ.1.1, XBB.1.5, and EG.5 (Figure 8C). Therefore, we propose that multivalent nanobodies have the ability to recognize multiple epitopes, thereby creating a larger contact area with the RBD and forming a spatial barrier that prevents ACE2 binding. Further, we compared the three nanobody‐recognized epitopes to ACE2‐recognized epitope characteristics (Figure 8E). the main recognition area of ACE2 and RBD is the notch area composed of Domain I/II/III, which has a wider action area. As mentioned before, ZF‐B11 mainly recognizes Domain I, while ZF‐E8 and SN‐F3 mainly recognize Domain I/II/III (Figure 8D), with minimal overlap among their epitopes. Therefore, B11‐E8‐F3 can recognize distinct epitopes on the same RBD or across different RBDs, enhancing its blocking efficacy. This may explain its broader spectrum and highly efficient neutralizing activity.

## 3 Discussion

Overall, this study reports a novel broad‐spectrum anti‐SARS‐CoV‐1/SARS‐CoV‐2 strategy with high scientific and applied value. The innovations and advantages are summarized as follows. First, nanobodies with longer CDR3 confer unique antigen recognition advantages, such as insertion into cavity epitopes or recognition of glycosylated epitopes.^[^ ^18^ ^]^ In addition, the nanobody and antigen binding surface are larger and are less likely to experience a reduction or loss of activity owing to virus mutation. In this study, the length of CDR3 of the three nanobodies, ZF‐B11, ZF‐E8, and SN‐F3, was 15, 16, and 34, respectively, which exceeds the length of CDR3 of conventional antibodies with 8–12 amino acids.^[^ ^19^ ^]^ In particular, SN‐F3, with an extra‐long CDR3 region, can form larger neck‐loop structures which makes it less likely for the virus to escape through mutation. Although it lost neutralizing activity for most of the Omicron sublineages in this study, it retained binding activity, which may confer a stronger spatial barrier to B11‐E8‐F3, resulting in a broader and more efficient neutralizing effect. Second, we propose a novel concept for the design of targeted nanobody libraries based on conserved epitope structures. Recently, de novo design of nanobody against specific epitope structural characteristics has been explored through de novo design.^[^ ^20^ ^]^ However, few reports are available on de novo antibody design, which may be due to the current low accuracy of antibody structure prediction, making it difficult to obtain candidate antibodies with high activity and druggability.^[^ ^21^ ^]^ Additionally, CDR3 are more diverse in conformation with different structures using similar sequences, thus physical modelling often relies on the experience of researchers. David et al. recently reported Nanobodies de novo design. The study is based on a neural network that was fine‐tuned and demonstrated that the RF diffusion network is capable of designing VHHs against specific epitopes from scratch by training thousands of experimentally determined antibody structures, as well as other real‐world experiments of antibody interactions.^[^ ^22^ ^]^ In contrast, this study designed the antigen–nanobody library with FR fixation and partial randomization in the CDR drawing on three conserved domain of pre‐Omicron S proteins and ACE2/S interaction information by de novo design. Among them, the FR was designed as a generic FR with high druggability and have some similarities with previously reported frameworks.^[^ ^23^ ^]^ But it is quite different from the traditional nanobody synthesis library in terms of CDR design, which is mainly derived from the structural characteristics of protein interactions between SARS‐CoV‐1/SARS‐CoV‐2 S protein and ACE2. Therefore, the de novo design of the nanobody library in this study was innovative and challenging. Third, nanobodies are small and flexible, and can easily be designed to recognize multiple epitopes. A multivalent targeting strategy has outstanding advantages when applied to viral infections, tumors and other diseases.^[^ ^24^ ^]^ Despite the COVID‐19 pandemic has come to an end, Omicron continues to mutate at a rapid frequency and escapes the vast majority of previously reported patient‐derived monoclonal antibodies.^[^ ^25^ ^]^ The monoclonal antibody SA55 as well as the bivalent nanobody n3130v are among the more widely reported SARS‐CoV‐2 inhibitors.^[^ ^26^ ^]^ However, it seems difficult to rely solely on monoclonal antibodies for broad‐spectrum antiviral efficacy as seen with SN‐F3 obtained in this study, even though it has a large area of recognition with the RBD and retains binding activity against the vast majority of Omicron sublineages, Omicron accumulates a large number of mutations on the RBD resulting in a significant conformational change in interaction with ACE2 leading to loss of its neutralizing activity. Although the multivalent design also does not fully guarantee its neutralizing effect on the subsequently generated SARS‐CoV‐2 mutants, at least the multivalent has multiple antigen recognition epitopes, which to some extent confers a more effective resistance to viral mutations. In contrast, the trivalent nanobody B11‐E8‐F3 we obtained in this study effectively neutralized the vast majority of pre‐EG.5 VOCs strains.

In this study, we carried out the design of a multivalent nanobody and retained activity, to some extent, against a variety of SARS‐CoV‐2 varaiants. Of note, these three nanobodies, when tandemly linked to form a trivalent nanobody, i.e., B11‐E8‐F3, exhibited the broadest‐spectrum and the most efficient antiviral activities. Furthermore, the trivalent nanobodies combine the respective advantages of the three nanobodies while counteracting the disadvantages. Taking ZF‐E8 and ZF‐B11 as examples, although ZF‐E8 could not completely cover the detection of VOCs pseudoviruses (10/15), their neutralization activity had IC_50_ values of less than 1 × 10^−9^ m against 90% of the variants, and this efficient neutralization activity was also directly conferred to B11‐E8‐F3. While ZF‐B11 almost completely covered all the detected VOCs pseudoviruses (15/15), so its broad spectrum was also well conferred to B11‐E8‐F3. Finally, we examined the preventive and therapeutic potential of B11‐E8‐F3 in three authentic virus animal models. Preventive administration is usually carried out in people at risk of viral exposure, whose viral load are relatively lower in the early stage, whereas therapeutic administration is usually carried out in people who are already infected, and may have obvious clinical symptoms, and a viral load that is usually very high. Thus, in the hACE2 transgenic mouse infection model, the viral dose used in the prevention model was 10‐fold lower than that in the therapeutic model. In this model, the lung viral load in the PBS group was 10‐fold (BA.5) and 100‐fold (XBB.1.16) lower than that of the therapeutic model on day 4, and the lung damage was also significantly less severe than that of the therapeutic model. In contrast, although the therapeutic administration was able to reduce lung viral load and lung inflammation to some extent, the treatment was less effective relative to preventive administration. This finding is similar to the clinical use of antibody drugs, which tends to be less effective in patients with mid‐ to late‐stage viral infections, as treatment of the ensuing storm of inflammatory factors and organ damage will be more challenging than controlling the virus. Therefore, it is reasonable to believe that B11‐E8‐F3 is a broad‐spectrum anti‐SARS‐CoV‐1/SARS‐CoV‐2 trivalent nanobody that covers the vast majority of currently reported VOCs, and that it demonstrates outstanding efficacy as a preventative. Therefore, B11‐E8‐F3 has an outstanding advantage in preventing SARS‐CoV‐1/SARS‐CoV‐2 infections, especially in immunocompromised populations or elderly people with high‐risk comorbidities.

## 4 Limitations

This study involved multiple methods to evaluate antibody activity, including binding activity, blocking ACE2/RBD activity, pseudovirus neutralization activity, and neutralization of authentic virus activity in vitro and in vivo. Thus, there are very few samples tested where the results in the two different experiments seem to be confusing, but the overall results are plausible and interpretable, and will be explained and discussed as follows. First, it should be stated that binding activity does not mean neutralizing activity, just as SN‐F3 can effectively bind Omicron sublineages but cannot effectively neutralize the Omicron pseudovirus. This is most likely because the RBD of the Omicron accumulates a large number of mutations, and although SN‐F3 still recognizes Omicron's RBD, there is a change from a pre‐Omicron blocking conformation (ACE2/RBD) to a non‐blocking conformation. And, it's worth noting that when we designed the library, the Delta strain was globally prevalent and Omicron was only just emerging, and thus we lacked the structural information on Omicron. SN‐F3 showed better neutralizing activity against the pre‐Omicron strain, supporting the credibility of our theoretical model. Second, ZF‐B11 has strong neutralization activity against SARS‐CoV‐2 (WT) pseudovirus but almost has no neutralization activity against authentic virus, which indirectly indicates that the infection mechanism of the pseudovirus cannot completely mimic the authentic virus. Third, the blocking activity of ACE2/RBD corresponds to their neutralizing activity in most cases, with some exception. For example, ZF‐B11 was not effective in blocking Delta's ACE2/RBD interactions, whereas it was effective against both pseudovirus and authentic virus, suggesting that it may affects virus‐cell fusion through structural deformation, as reported in previous studies.^[^ ^27^ ^]^ Finally, the neutralizing activity of B11‐E8‐F3 was only tested in vitro and in mouse models. Further clinical studies are necessary to determine the drug's safety, as well as its preventive and therapeutic effects.

## 5 Experimental Section

Key information of reagents and resources are shown in Table S6 (Supporting Information).

### HIV Pseudovirus

The S protein sequences of SARS‐CoV‐1, SARS‐CoV‐2, and its variants were retrieved from the Global Initiative on Sharing All Influenza Data (GISAID) database, and were synthesized, and cloned into the pCDNA3.4 vector, respectively. All S proteins were truncated by 19 amino acids at the C‐terminus to increase packaging efficiency, which sequences are listed in Table S7 (Supporting Information). Detailed methods for packaging HIV pseudoviruses have been described previously.^[^ ^28^ ^]^ Briefly, pSG3^Δenv^ and recombinant vector were cotransfected into HEK293T cells. The supernatants were collected after 48 h post‐transfection, aliquoted, and stored at ‒80 °C. HEK293T(ACE2‐OE) cells were infected with 10‐fold serially diluted pseudoviruses, and the infection value was measured using a luciferase assay kit (Beyotime). The positive cutoff value was set at 5‐fold more than that of the mock infection control. The viral titer was calculated using the Reed–Muench method.

### VSV Pseudovirus

The VSV‐based replication‐competent pseudovirus was rescued using a modified method as previously reported.^[^ ^29^ ^]^ Briefly, the VSV‐G gene cassette in the pVSV‐GFP plasmid was replaced with SARS‐CoV‐2 S∆21 to make a pVSV‐∆G‐SARS‐CoV‐2 S‐GFP plasmid. Recombinant VSV virus was rescued co‐transfecting HEK293T/VeroE6 mixture cells with pVSV‐∆G‐SARS‐CoV‐2 S‐GFP, and N, P, L, and VSV‐G helper plasmids and the T7 polymerase expression plasmid. Successful rescue of the recombinant virus was monitored by visualizing GFP protein expression. The same method was used to rescue recombinant VSV‐∆G‐SARS‐CoV‐2 S NanoLuc viruses. Successful rescue of NanoLuc viruses was monitored by measuring the nanoluciferase activity using a commercial kit (Promega). Rescued viruses were propagated and titrated using VeroE6 cells. The viral titer was calculated using the Reed–Muench method. For rescuing the WT and Deltapseudoviruses, deletion of the C‐terminal 21 amino acid of the S protein was conducted.

### Authentic Virus

C57M14 is an adapted virus by repeated infection and passage of the SARS‐CoV‐2 (WT) virus in BALB/c mice.^[^ ^30^ ^]^ We demonstrated that C57M14 can fully represent the SARS‐CoV‐2 (WT) prototype strain because deep sequencing revealed that C57M14 has only one mutation site on the S protein, namely Q498H(S). Notably, C57M14 is a lethal strain that can cause death in BALB/c mice at specific doses. Delta, BA.2, BA.5 XBB.1.16 and EG.5 were isolated from infected donors. All procedures involving authentic viruses were conducted in a Biosafety Level 3 laboratory (Key Laboratory of Jilin Province for Zoonosis Prevention and Control, Changchun, China).

### Cells and Cell Culture

HEK293T, HEK293T(ACE2‐OE) and Vero E6 cells were cultured in DMEM (Gibco) supplemented with 10% FBS (Gemini). CHO‐S cells were cultured in Expi CHO Expression Medium (Gibco). HEK293T(ACE2‐OE) is HEK293T transfected with ACE2 overexpression.

### Animals

One‐year‐old unimmunized female camels were selected for antigenic immunization. Mice and Syrian golden hamsters were maintained under specific pathogen‐free conditions and all experimental procedures involving these animals were approved by the Laboratory Animal Welfare and Ethics Committee of the Changchun Veterinary Research Institute, Chinese Academy of Agricultural Sciences (IACUC AMMS‐11‐2023‐033).

### Nanobody Library Construction and Screening—De Novo Design of a Synthetic Nanobody Library based on Structure‐Specific Features

Nanobody sequences were collected from a web database (https://sdab‐db.ca) and analyzed using the multiple‐sequence alignment ClustalW program. The nanobody FR sequences were compared and analyzed with the human VH3 FR sequences, the same sequences were retained, and for the inconsistent sequences, the FR and human VH3 frameworks were converged as much as possible, taking into account the conformational stability and immunogenicity. Finally, universal framework sequences including FR1, FR2, FR3, and FR4 with stable conformation, high effectiveness, and high expression were designed and modelled by Insight II (MSI Corporation, San Diego). Subsequently, based on the 3‐D complex structures of ACE2 and SARS‐CoV‐1, SARS‐CoV‐2, or its variants (including Alpha, Beta, Gamma, and Delta) from the PDB, the interaction‐binding domain and key amino acid residues were studied. Considering the frequency and length of the CDR amino acid distribution, a series of positive mutant residues was introduced into CDR1, CDR2, and CDR3. Subsequently, a set of de novo designed, fully synthetic nanobody libraries were obtained; that is, the nanobody framework regions (FR1, FR2, FR3, and FR4) were fixed, and CDR (CDR1, CDR2 and CDR3) was partially positively randomized. Finally, using reverse translation and codon optimization methods, trimer primers design and overlapping PCR were performed in the construction of nanobody synthetic library. Of note, the nanobody synthetic library is synthesized by designing trimer primers, i.e., trimer phosphoramidites formed by linking three nucleosides in a predetermined type and order, and these different triple nucleosides correspond to the encoded amino acids one by one. The trimer primer design and overlap PCR splicing were used to form the nanobody DNA library, which was synthesized by Suzhou GENEWIZ Biotechnology Co., Ltd. and constructed into the pComb3XTT phage display vector.

### Camel Immunization and Library Construction

Omicron RBD protein was used to immunize a naïve Bactrian camel in six immunizations at 7‐day intervals. The protein was mixed with Freund's complete adjuvant for the first two immunizations and mixed with Freund's incomplete adjuvant for the subsequent immunizations. Venous blood was collected prior to the fifth immunization for serum nanobody titer measurement using an indirect enzyme‐linked immunosorbent assay (ELISA). On the 38^th^ day, 200 mL peripheral blood was collected for nanobody library construction. The detailed immunization schedule is shown in Table S1 (Supporting Information). After the isolation of peripheral blood lymphocytes from immunized camels, RNAs was extracted and transcribed into cDNA, and the nanobodies were amplified using two‐step nested PCR.^[^ ^31^ ^]^ Purified nanobody DNA fragments were cloned into the immobilized phage display phagemid pComb3XTT.

### Phage Display and Screening

The recombinant nanobody library plasmids were electroporated into TG1 electrocompetent cells, which were further infected with the M13k07 helper phage to prepare phage display nanobody libraries. The quality of the libraries was evaluated using electrotransfer library capacity and sequence accuracy. After establishing the library, 100 clones were randomly selected for sequencing. Sequence accuracy was determined by calculating the ratio of the correct sequence to the total sequence. Nanobodies specific for the SARS‐CoV S and SARS‐CoV‐2 S proteins, and Omicron RBD were selected from the phage display library and enriched through three consecutive rounds of biopanning. Colonies were picked from the enriched pool and positive clones were identified using indirect phage ELISA.

### Protein Expression

Monovalent nanobodies were constructed using human IgG1 Fc and a hinge forming a symmetrical structure. For the design of multivalent nanobodies, the bivalent and trivalent nanobodies were linked through a length of 10 (G_4_S)_2_ and 15 (G_4_S)_3_ amino acids, respectively, and a 6× His purification tag was introduced at the C‐terminus of each. The coding sequences were cloned into the pcDNA3.4 eukaryotic vector. The recombinant plasmids were transfected into CHO‐S cells in the logarithmic growth phase. Supernatants were collected after seven days and further purified through Protein A or Ni^2+^ chromatography. Protein purity and concentration were determined through HPLC‐SEC and the bicinchoninic acid assay.

### ELISA—Indirect ELISA

Microplates were coated with of RBD or S protein (1 µg mL^−1^, 100 µL) in 0.05 m carbonate‐buffered saline, blocked with 4% (w/v) skim milk, and incubated with VHH‐hFc candidates and 1:3000 horseradish peroxidase labelled goat anti‐human secondary antibody (GAH‐HRP). The microplate was washed three times with PBST after every incubation. The absorbance at 450 nm was determined after the development of color in the colorimetric reaction.

### Competitive ELISA

To determine the epitope crossover between the recognition RBD of the three nanobodies and the competition for the ACE2/RBD complex, microplates were coated with RBD or S protein (1 µg mL^−1^) in 0.05 m carbonate‐buffered saline, blocked with 4% skim milk. For the identification of cross‐recognition epitopes between antibodies, VHH‐Fc (100 µg mL^−1^) or B11‐E8‐F3 (100 µg mL^−1^) and biotin‐labelled VHH‐Fc (1 µg mL^−1^) were premixed and added to the corresponding wells on the plates. For the identification of antibodies competing for ACE2/RBD protein interactions, serially diluted VHH‐Fc or B11‐E8‐F3 of the gradient and biotin‐labelled ACE2 (1 µg mL^−1^) were premixed and added to the corresponding wells. Streptavidin‐HRP was added as the secondary antibody. Absorbance at 450 nm was determined after the development of color in the colorimetric reaction. The percentage of inhibition was calculated through the percentage of reduction of OD_450 nm_ compared to the OD_450 nm_ of the control well (biotin‐labelled‐antibody‐only), according to the following equation

(1) $$\begin{array}{ccc} & & \mathrm{Inhibition}\;(\%)\\ & & \;=\frac{\mathrm{Control}\;\mathrm{well}\;\left(\mathrm{OD}450\mathrm{nm}\right)-\mathrm{Test}\;\mathrm{well}\left(\mathrm{OD}450\mathrm{nm}\right)}{\mathrm{Control}\;\mathrm{well}\left(\mathrm{OD}450\mathrm{nm}\right)}\times 100\% \end{array}$$

### Affinity Assays by Biolayer Interferometry (BLI)

Affinity assays were performed on ForteBio Octet RED96e System (Sartorius). Biotinylated‐B11‐E8‐F3 (10 µg/mL) was coupled to SA Biosensor (Sartorius). RBD variant proteins were diluted from 500 × 10^−9^ to 7.81 × 10^−9^ M, and the association and dissociation was set at 120s and 180 s, respectively. The data were analyzed using a 1:1 binding model.

### Neutralization Assays—Neutralization Assay Against HIV Pseudoviruses

Serially diluted nanobodies (50 µL) were mixed with an equal volume of pseudovirus in a 96‐well plate for 1 h at 37 °C as previously described, and coincubated with 2 ×10^4^/well HEK293T(ACE2‐OE) cells for 48 h. Luciferase activity was evaluated measuring relative luminescence units (RLU), and the percentage inhibition was calculated as the percentage reduction of RLU compared to the control (pseudovirus‐only) as follows

(2) $$\begin{array}{c} \mathrm{Inhibition}\;\left(\%\right)=\frac{\mathrm{Control}\;\mathrm{well}\;\left(\mathrm{RLU}\right)-\mathrm{Test}\;\mathrm{well}\left(\mathrm{RLU}\right)}{\mathrm{Control}\;\mathrm{well}\left(\mathrm{RLU}\right)}\times 100\% \end{array}$$

### Neutralization Assay Against VSV Pseudo viruses

Vero E6 cells (1×10^5^/well) were seeded into 24‐well plates 20 h before infection. Nanobodies were diluted and incubated with an equal volume of VSV‐∆G‐SARS‐CoV‐2 S‐GFP pseudovirus (WT, Delta, and Omicron) for 1 h at 37°C. The virus used for infection was at 2500 50% tissue culture infectious dose (TCID_50_) per well. The antibody–virus mixture (500 µL) was added directly to Vero E6 cells and incubated for 1 h. The plate was rotated every 15 min to ensure equal distribution of the virus. One‐hour post‐infection, the inoculated virus was removed, and the cells were washed with PBS and fed fresh complete MEM. The virus‐infected cells were cultured for 36 h. Viral infection was monitored by visualizing the GFP expression using a fluorescence microscopy. The wells were imaged using fluorescence microscopy when the virus‐only control wells were nearly 100% positive.

### Neutralization Assay Against Authentic Viruses

Vero E6 cells (1×10^4^/well) were prepared in 96‐well plates 20 h before infection. Diluted antibodies (200 µL) were mixed with an equal volume of authentic virus (SARS‐CoV‐2 (WT), Delta, BA.2, BA.5, XBB.1.16, and EG.5) with 200 TCID_50_; 100 µL mixture was added and incubated with Vero E6 cells for 72 h. CPE in each well was recorded under a microscope.

### Animal Assays—Pseudoviral Infection Model: VSV Pseudoviruses

Hamsters were intraperitoneally injected with nanobodies (10 mg kg^−1^) or isotype control antibodies and 24 h afterward were intranasally infected with the pseudovirus. The infection dose per hamster was 6 × 10^5^, 2.5 × 10^6^, and 3 × 10^5^ TCID_50_, for the WT, Delta, and Omicron viruses, respectively. The pseudovirus used for the infection of the hamsters contained a secreted NanoLuc reporter for easy monitoring of the virus infection with a blood draw. The first blood sample was collected 12 h post‐infection, followed by a second and a third blood sample at 12 h intervals. Further samples were collected every 24 h from the WT and Omicron pseudovirus‐infected hamsters. The first blood collection was performed 24 h post‐infection, and subsequent blood collection was performed every 24 h for Delta‐pseudovirus‐infected hamsters. Serum was isolated and diluted with PBS to measure nanoluciferase activity. Hamsters were bioluminescently imaged under anesthesia at 24 h post‐infection for WT and Omicron pseudovirus, and 48 h post‐infection for Delta pseudovirus. The nanoluciferase substrate furimazine was diluted with PBS and injected via the orbital sinus vein. The bioluminescence signal was acquired immediately after the injection of furimazine for 5 min continuously using IVIS Imaging Systems software (PerkinElmer).

### Authentic Virus Infection Model—Prevention Assay

Eight‐week‐old female BALB/c mice were housed in an SPF‐grade animal house for 7‒10 days. Then, 30 mice were randomly assigned into three groups (*n* = 10): high‐dose (HD, 280 µg), low‐dose (LD, 50 µg), and negative (PBS) groups. The drug was administered through nasal drops 6 h in advance, after which the authentic virus (C57M14, 10^4^ TCID_50_) was inoculated through nasal drops. Body weight was monitored daily. On the third and sixth days, three mice were euthanized, and their turbinate bone tissue and half of the lung tissue was examined for viral load using RT‐qPCR; the other half of the lung tissue was used for HE staining to observe lung pathological changes.

Human ACE2 transgenic C57BL/6J mice (Shanghai Model Organisms Centre) were inoculated intranasally with BA.5 (10^3^ TCID_50_) or XBB.1.16 (10^1.75^ TCID_50_) and randomly divided into four groups (*n* = 5), then mice were intranasally administered 280 µg B11‐E8‐F3 or an equal volume of PBS 2 h before viral challenge. On the fourth day, all mice were euthanized, lung tissue was obtained and analyzed as previously described.

### Therapeutic Assay

For the therapeutic assay, eight‐week‐old female BALB/c mice (Beijing Vitalstar Biotechnology) were randomly assigned into three groups (*n* = 10): therapeutic group 1 (TP1, 280 µg, 6/30 h), therapeutic group 2 (TP2, 280 µg, 24/48 h), and negative control group (PBS). First, a viral dose (C57M14 10^4^ TCID_50_) was administered through nasal drops. The TP1 group was administered 280 µg B11‐E8‐F3 through nasal drops after 6 and 30 h post viral challenge. The TP2 group was administered 280 µg B11‐E8‐F3 through nasal drops after 24 and 48 h post viral challenge. Body weight was monitored daily. On the third and sixth days, three mice were euthanized, and their turbinate bone tissue and half of the lung tissue were collected and analyzed as described before.

Human ACE2 transgenic C57BL/6J mice were inoculated intranasally with BA.5 (10^4^ TCID_50_) or XBB.1.16 (10^2.75^ TCID_50_), randomly divided into four groups (*n* = 4), and intranasally administered with 280 µg B11‐E8‐F3 or an equal volume of PBS 2, 26, and 50 h after viral challenge. On the fourth day, all mice were euthanized, lung tissues were obtained and analyzed as previously described.

It is worthy nothing that, in the BALB/c infection model, the LD group in prevention assay and two therapeutic groups assays are conducted simultaneously, and therefore shared the same PBS as control. And the HD therapeutic group was done independently, therefore a separate PBS control group was set.

### Computer‐Aided Structural Model and Docking

The 3D spatial structure of ZF‐B11, ZF‐E8 and SN‐F3 were modeled by computer‐guided homology modeling method, and its free energy is minimized by molecular dynamics method based on InsightII 2000 software under Sun workstation. The crystal structure of SARS‐CoV‐2(WT) RBD, ACE2&RBD were obtained from Protein Data Bank (PDB code: 7Q3R, 7WHH) and further optimized to fine structures. The binding complex theoretical structure of nanobodies and RBD were constructed using molecular docking method, and optimized using molecular dynamics methods. All the structures were refined using DPtechnology's Hermite platform.

### Statistical Analysis

All the quantitative data were presented as mean ± standard deviation (SD) with at least two independent experiments. EC50 or IC50 was calculated through four‐parameter regression using GraphPad Prism (v.9.4.1). Detailed sample sizes (*n*) were labeled in the figures or figure legends. The two‐tailed Student's *t*‐test and Mann–Whitney test were used to determine the statistical significance between groups as showed in the figure legends. The statistical significance was indicated as * *p* < 0.05, ** *p* < 0.01 and ***p < 0.001, 95% confidence level, using IBM SPSS (v.27.0.1).

## Conflict of Interest

The authors declare no conflict of interest.

## Supporting information



Supporting Information

## Data Availability

The data that support the findings of this study are available from the corresponding author upon reasonable request.
