# Supplementary material for: Broad‐Spectrum Engineered Multivalent Nanobodies Against SARS‐CoV‐1/2
Source: Adv Sci (Weinh). 2024 Oct 7;11(45):2402975. doi: 10.1002/advs.202402975 (PMC11615778; doi:10.1002/advs.202402975)
Supplement: Supplementary file 1 — Supporting Information [file ADVS-11-2402975-s001.pdf]

ADVANCED SCIENCE
Open Access

Supporting Information

for *Adv. Sci.*, DOI 10.1002/advs.202402975

Broad-Spectrum Engineered Multivalent Nanobodies Against SARS-CoV-1/2

Zhihong Wang, Zhuangzhuang Shi, Xiaochen Liao, Guiqi Quan, Hui Dong, Pinnan Zhao, Yangyihua Zhou, Ning Shi, Jie Wang, Yahui Wu, Chunxia Qiao, Xin ying Li, Ran Zhang, Zekun Wang, Tiecheng Wang*, Xiang Gao*, Jiannan Feng* and Longlong Luo**

Figure S1

A

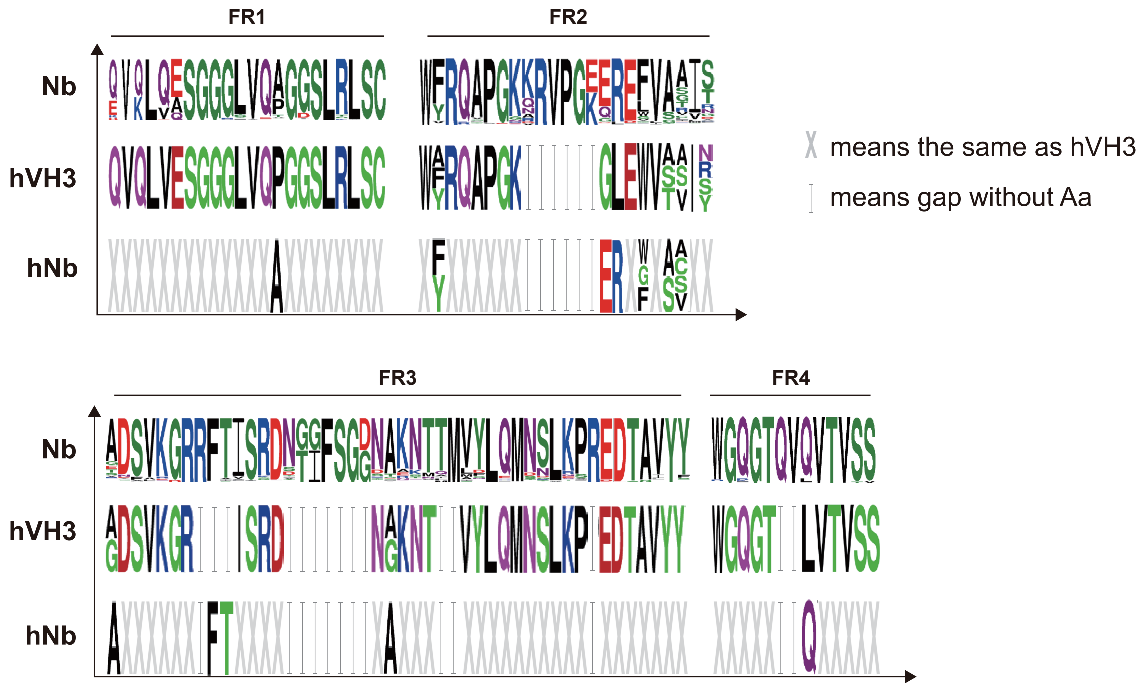

B

QVQLVESGGGLVQAGGSLRLSCAASGF(R/S/N)TF(V/S)S(D/Y/N)S(R/D/N/H)I(Y/S/N)Y(N/S)AMGWY(F)RQAPGKEREW(G/F)VA(S)A(C/S/V)IS(Y/N/R)S(W/T/R/N)DGS(N/R/G)G(D/S/Y)S(R/G/N)G(D/T/Y)S(N/Y/R/I/H)TY(N/S)YADSVKGRFTISRDNAKNTVYLQMNSLKPEDTAVYYCAAD(R/G/S)P(R/S/D/A)G(Y/W/S/R)T(S/V/P/W/Y)Y(S/R/V)V(Y/R/S)Y(W/T/H)VKP(S/V/N)DS(Y/E/R)GS(E/Q/Y)S(Y/R/F)G(D/N/R)Y(H/D/Q)R(G/S)Y(A/R/L)Y(S/E/N)Y(G/W/F)P(S/Y/C)L(S/Y/R)W(C/S/R)P(S/Y/D)E(S/V)R(G/D)DYDYWGQGTQVTVSS

C

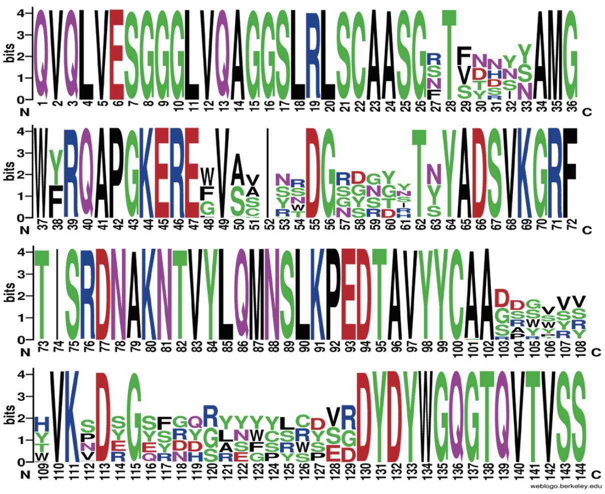

Fig. S1: Construction of fully human nanobody synthetic library.
(A) Multiple sequence alignment of FRs between nanobody (Nb), human VH3 (hVH3) and human nanobody (hNb). In the Nb sequence alignment, the height of the amino acid represents its frequency of occurrence at the site.
(B) Amino acid sequence of the fully human nanobody synthetic library. The FRs of the nanobodies are indicated by different highlighted colours: red for FR1, green for FR2, purple for FR3, and dark blue for FR4. The unmarked coloured regions are in the order of CDR1/2/3, where F(V/S) indicates that the amino acids F, V, and S occur at the same frequency at that site.
(C) Multiple sequence alignment of the fully human nanobody library construction. The occurrence of multiple amino acids at certain sites represents the variety of amino acids at that site, while the frequency of occurrence of each amino acid is positively correlated with its height.

Figure S2

A

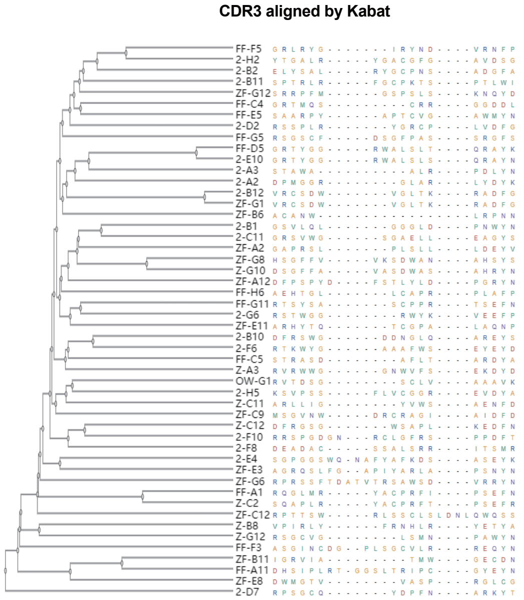

B

Delta	Z-C11	2-H5	FF-A11	FF-E5	Z-C2	ZF-E11	2-B11	ZF-E8	2-H2	2-A3
	ZF-B11	FF-F3	Z-G12	ZF-G1	2-B12	OW-G11	ZF-E3	FF-C4	FF-F5	ZF-A12
	ZF-G12	2-D2	Z-G10	ZF-G6	2-C11	FF-G5	ZF-A2	ZF-G8	2-D7	2-E10
	2-G6	FF-G11	Z-A3	ZF-B6	2-A2	2-E4	FF-D5	2-B2	FF-H6	Z-B8
	ZF-C12	2-B1	2-F6	2-F10	FF-A1	FF-C5	Z-C12	ZF-C9	2-B10	2-F8
Omicron	Z-C11	2-H5	FF-A11	FF-E5	Z-C2	ZF-E11	2-B11	ZF-E8	2-H2	2-A3
	ZF-B11	FF-F3	Z-G12	ZF-G1	2-B12	OW-G11	ZF-E3	FF-C4	FF-F5	ZF-A12
	ZF-G12	2-D2	Z-G10	ZF-G6	2-C11	FF-G5	ZF-A2	ZF-G8	2-D7	2-E10
	2-G6	FF-G11	Z-A3	ZF-B6	2-A2	2-E4	FF-D5	2-B2	FF-H6	Z-B8
	ZF-C12	2-B1	2-F6	2-F10	FF-A1	FF-C5	Z-C12	ZF-C9	2-B10	2-F8
BA.2	Z-C11	2-H5	FF-A11	FF-E5	Z-C2	ZF-E11	2-B11	ZF-E8	2-H2	2-A3
	ZF-B11	FF-F3	Z-G12	ZF-G1	2-B12	OW-G11	ZF-E3	FF-C4	FF-F5	ZF-A12
	ZF-G12	2-D2	Z-G10	ZF-G6	2-C11	FF-G5	ZF-A2	ZF-G8	2-D7	2-E10
	2-G6	FF-G11	Z-A3	ZF-B6	2-A2	2-E4	FF-D5	2-B2	FF-H6	Z-B8
	ZF-C12	2-B1	2-F6	2-F10	FF-A1	FF-C5	Z-C12	ZF-C9	2-B10	2-F8
BA.2.12.1	Z-C11	2-H5	FF-A11	FF-E5	Z-C2	ZF-E11	2-B11	ZF-E8	2-H2	2-A3
	ZF-B11	FF-F3	Z-G12	ZF-G1	2-B12	OW-G11	ZF-E3	FF-C4	FF-F5	ZF-A12
	ZF-G12	2-D2	Z-G10	ZF-G6	2-C11	FF-G5	ZF-A2	ZF-G8	2-D7	2-E10
	2-G6	FF-G11	Z-A3	ZF-B6	2-A2	2-E4	FF-D5	2-B2	FF-H6	Z-B8
	ZF-C12	2-B1	2-F6	2-F10	FF-A1	FF-C5	Z-C12	ZF-C9	2-B10	2-F8
BA.4	Z-C11	2-H5	FF-A11	FF-E5	Z-C2	ZF-E11	2-B11	ZF-E8	2-H2	2-A3
	ZF-B11	FF-F3	Z-G12	ZF-G1	2-B12	OW-G11	ZF-E3	FF-C4	FF-F5	ZF-A12
	ZF-G12	2-D2	Z-G10	ZF-G6	2-C11	FF-G5	ZF-A2	ZF-G8	2-D7	2-E10
	2-G6	FF-G11	Z-A3	ZF-B6	2-A2	2-E4	FF-D5	2-B2	FF-H6	Z-B8
	ZF-C12	2-B1	2-F6	2-F10	FF-A1	FF-C5	Z-C12	ZF-C9	2-B10	2-F8
BA.5	Z-C11	2-H5	FF-A11	FF-E5	Z-C2	ZF-E11	2-B11	ZF-E8	2-H2	2-A3
	ZF-B11	FF-F3	Z-G12	ZF-G1	2-B12	OW-G11	ZF-E3	FF-C4	FF-F5	ZF-A12
	ZF-G12	2-D2	Z-G10	ZF-G6	2-C11	FF-G5	ZF-A2	ZF-G8	2-D7	2-E10
	2-G6	FF-G11	Z-A3	ZF-B6	2-A2	2-E4	FF-D5	2-B2	FF-H6	Z-B8
	ZF-C12	2-B1	2-F6	2-F10	FF-A1	FF-C5	Z-C12	ZF-C9	2-B10	2-F8

OD450(nm)
>3
>2
>1
<1

C

B.1.351	***Z-C11***	2-H5	FF-A11	FF-E5	Z-C2	ZF-E11	2-B11	***ZF-E8***	2-H2	2-A3
	ZF-B11	FF-F3	Z-G12	***ZF-G1***	2-B12	***OW-G11***	***ZF-E3***	FF-C4	FF-F5	ZF-A12
	ZF-G12	2-D2	***Z-G10***	ZF-G6	***2-C11***	FF-G5	ZF-A2	ZF-G8	2-D7	2-E10
	2-G6	FF-G11	Z-A3	ZF-B6	***2-A2***	2-E4	***FF-D5***	***2-B2***	FF-H6	***Z-B8***
	ZF-C12	2-B1	2-F6	2-F10	FF-A1	FF-C5	Z-C12	ZF-C9	2-B10	2-F8

Neutralization (%)
>40%
>20%
>0%
<0%

BA.2	***Z-C11***	2-H5	FF-A11	FF-E5	Z-C2	ZF-E11	2-B11	***ZF-E8***	2-H2	2-A3
	ZF-B11	FF-F3	Z-G12	***ZF-G1***	2-B12	***OW-G11***	***ZF-E3***	FF-C4	FF-F5	ZF-A12
	ZF-G12	2-D2	***Z-G10***	ZF-G6	***2-C11***	FF-G5	ZF-A2	ZF-G8	2-D7	2-E10
	2-G6	FF-G11	Z-A3	ZF-B6	***2-A2***	2-E4	***FF-D5***	***2-B2***	FF-H6	***Z-B8***
	ZF-C12	2-B1	2-F6	2-F10	FF-A1	FF-C5	Z-C12	ZF-C9	2-B10	2-F8

Neutralization (%)
>60%
>40%
>20%
<20%

Fig. S2: Screening of nanobody candidates.

(A) Alignment of 50 representative sequences selected from 163 positive clones according to the similarity of their CDR3 sequences using Kabat.

(B) Binding activity of 50 nanobody candidate supernatants to RBD proteins of SARS-CoV-2 variants (Delta, Omicron, BA.2, BA.2.12.1, BA.4, and BA.5) by ELISA (n=2).

(C) Neutralizing activity of 50 nanobody candidate supernatants against HIV pseudoviruses of SARS-CoV-2 variants (B.1.351 and BA.2) evaluated as described previously (n=3).

Figure S3

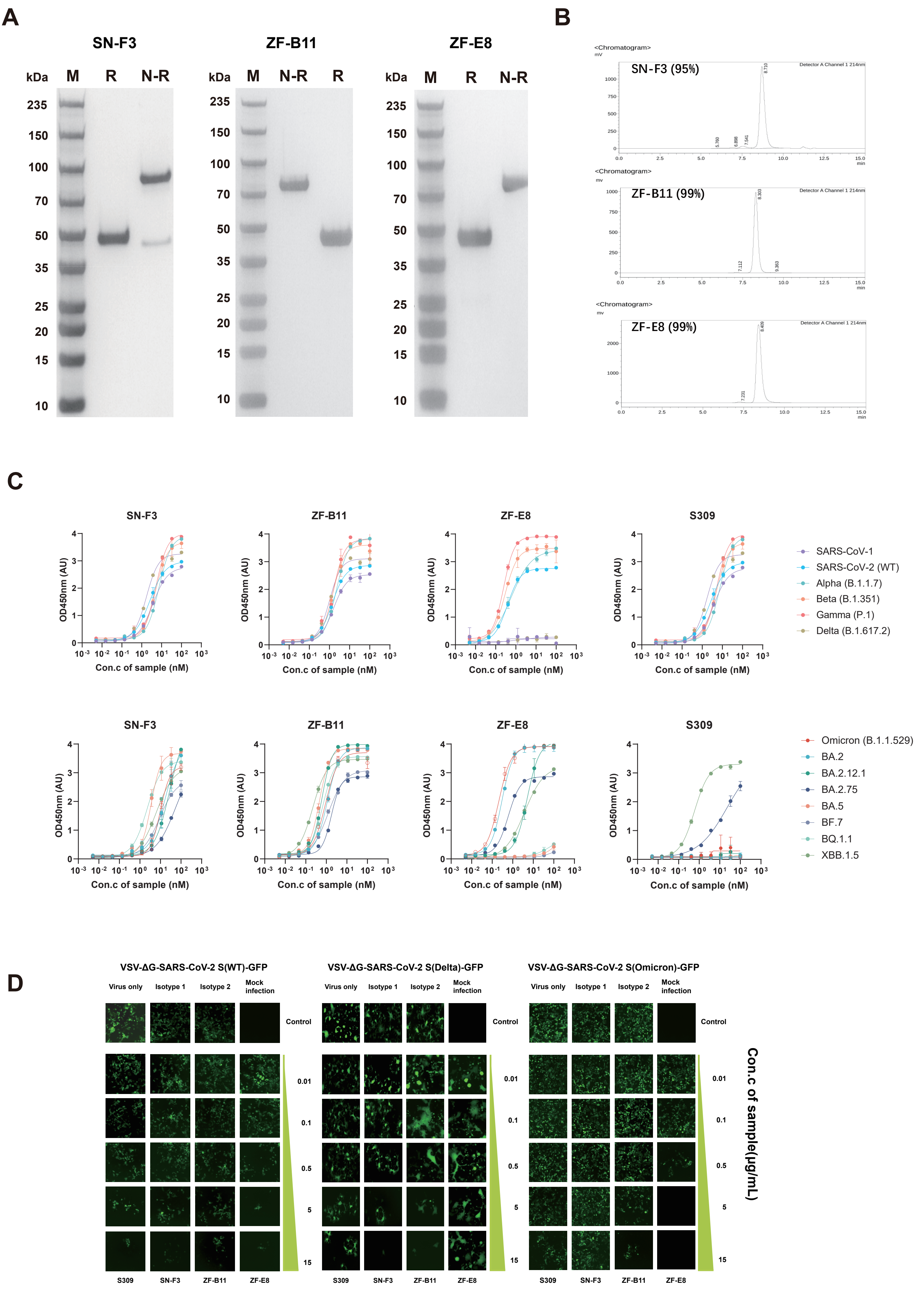
A
SN-F3
ZF-B11
ZF-E8
kDa
M
R
N-R
235
150
100
70
50
35
25
20
15
10
B
SN-F3 (95%)
ZF-B11 (99%)
ZF-E8 (99%)
Detector A Channel 1 214nm
C
SN-F3
ZF-B11
ZF-E8
S309
OD450nm (AU)
Con.c of sample (nM)
SARS-CoV-1
SARS-CoV-2 (WT)
Alpha (B.1.1.7)
Beta (B.1.351)
Gamma (P.1)
Delta (B.1.617.2)
Omicron (B.1.1.529)
BA.2
BA.2.12.1
BA.2.75
BA.5
BF.7
BQ.1.1
XBB.1.5
D
VSV-ΔG-SARS-CoV-2 S(WT)-GFP
VSV-ΔG-SARS-CoV-2 S(Delta)-GFP
VSV-ΔG-SARS-CoV-2 S(Omicron)-GFP
Virus only
Isotype 1
Isotype 2
Mock infection
Control
0.01
0.1
0.5
5
15
S309
SN-F3
ZF-B11
ZF-E8
Con.c of sample(μg/mL)

E

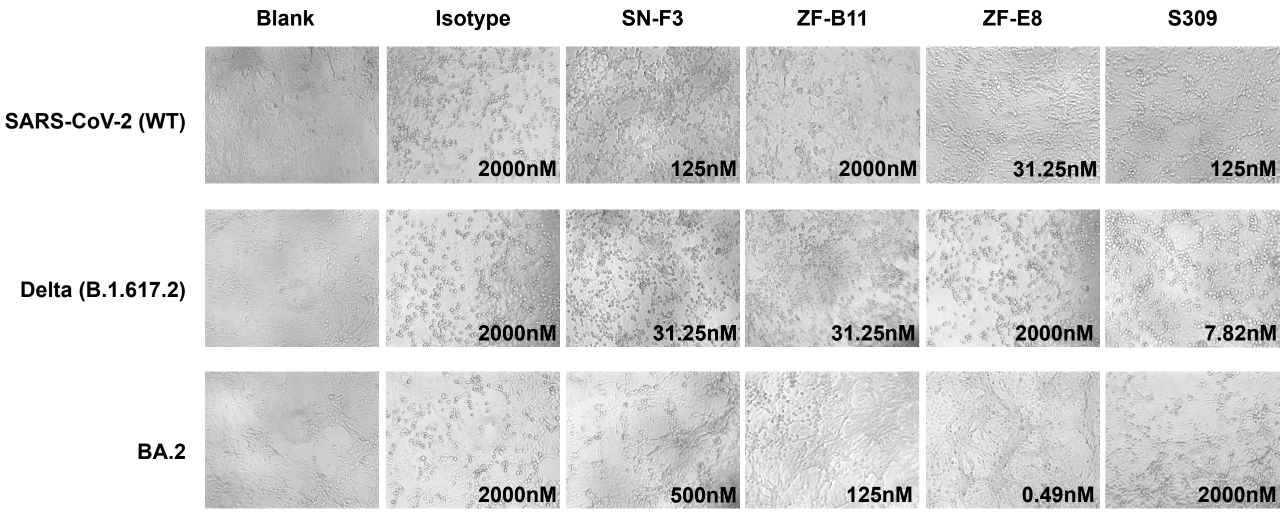

Fig. S3: Expression and functional verification of SN-F3, ZF-B11, and ZF-E8.

(A) SDS-PAGE of SN-F3, ZF-B11, and ZF-E8 in reduced (channel R) and non-reduced (channel N-R) states.

(B) HPLC analysis of SN-F3, ZF-B11, and ZF-E8.

(C) Binding activity of SN-F3, ZF-B11, and ZF-E8 to 15 S/RBD proteins of SARS-CoV-1 and SARS-CoV-2 VOCs/VOIs (n=2).

(D) Neutralizing activity of SN-F3, ZF-B11, and ZF-E8 against three VSV-GFP pseudoviruses (WT/Delta/Omiron) (200×). Isotope 1 is a clinically investigational anti-ricin antibody drug developed in our laboratory. Isotype 2 is a commercialized anti-HER2 antibody drug Herceptin.

(E) Neutralizing activity of SN-F3, ZF-B11, and ZF-E8 against three SARS-CoV-2 authentic viruses (WT/Delta/BA.2).

Figure S4

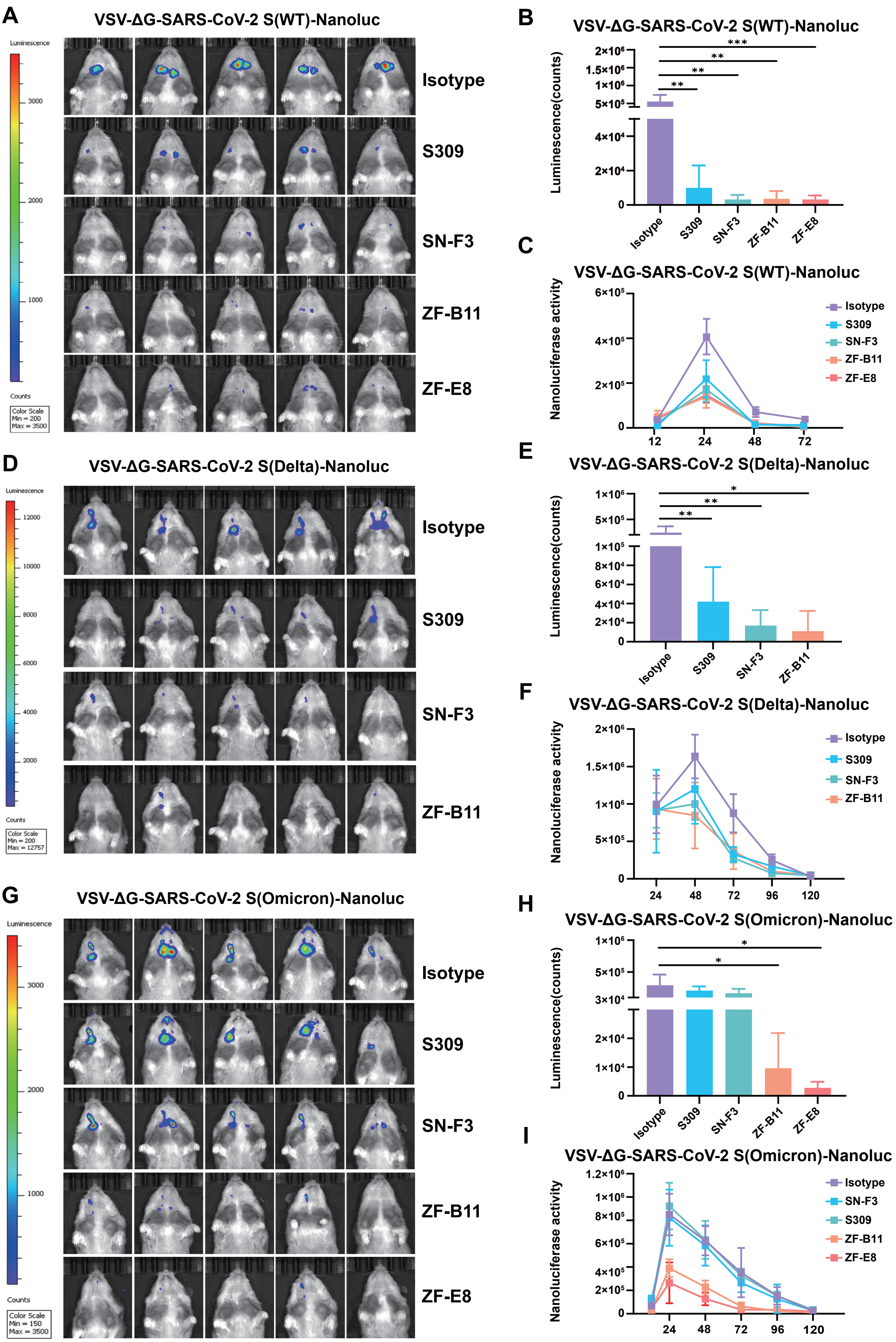
A
VSV-ΔG-SARS-CoV-2 S(WT)-Nanoluc
Isotype
S309
SN-F3
ZF-B11
ZF-E8
Color Scale
Min = 200
Max = 3500
B
VSV-ΔG-SARS-CoV-2 S(WT)-Nanoluc
Luminescence(counts)
Isotype
S309
SN-F3
ZF-B11
ZF-E8
C
VSV-ΔG-SARS-CoV-2 S(WT)-Nanoluc
Nanoluciferase activity
D
VSV-ΔG-SARS-CoV-2 S(Delta)-Nanoluc
Color Scale
Min = 200
Max = 12757
E
VSV-ΔG-SARS-CoV-2 S(Delta)-Nanoluc
F
VSV-ΔG-SARS-CoV-2 S(Delta)-Nanoluc
G
VSV-ΔG-SARS-CoV-2 S(Omicron)-Nanoluc
Color Scale
Min = 150
Max = 3500
H
VSV-ΔG-SARS-CoV-2 S(Omicron)-Nanoluc
I
VSV-ΔG-SARS-CoV-2 S(Omicron)-Nanoluc

Fig. S4: Neutralizing activity of SN-F3, ZF-B11, and ZF-E8 against VSV-ΔG-SARS-CoV-2 S (WT/Delta/Omicron)-NanoLuc in Syrian golden hamsters.

All studies were conducted in Syrian golden hamsters treated with antibodies at a dose of 10 mg/kg intraperitoneally and challenged with an intranasal inoculation of VSV-ΔG-SARS-CoV-2 S (WT/Delta/Omicron)-NanoLuc with 6×10^5 TCID50 for WT virus, 2.5×10^6 TCID50 for Delta virus, and 3×10^5 TCID50 for Omicron virus, respectively, after 24 h (n=5).

(A, D, and G) Localization of VSV-ΔG-SARS-CoV-2 S (WT/Delta/Omicron)-NanoLuc in hamsters after pseudovirus challenge, visualised by IVIS Imaging Systems, including WT (A, 24 h), Delta (D, 48 h) and Omicron (G, 24 h).

(B, E, and H) Quantified fluorescence intensity of VSV-ΔG-SARS-CoV-2 S (WT/Delta/Omicron)-NanoLuc using Image J, including WT (B, 24 h), Delta (E, 48 h) and Omicron (H, 24 h). Statistical significance was analysed using one-way ANOVA. ns, no significance; $*P < 0.05$; $**P < 0.01$; $***P < 0.001$.

(C, F, and I) Serum concentration of VSV-ΔG-SARS-CoV-2 S (WT/Delta/Omicron)-NanoLuc at different time points measured as nanoluciferase activity, including WT (C), Delta (F), and Omicron (I).

Figure S5

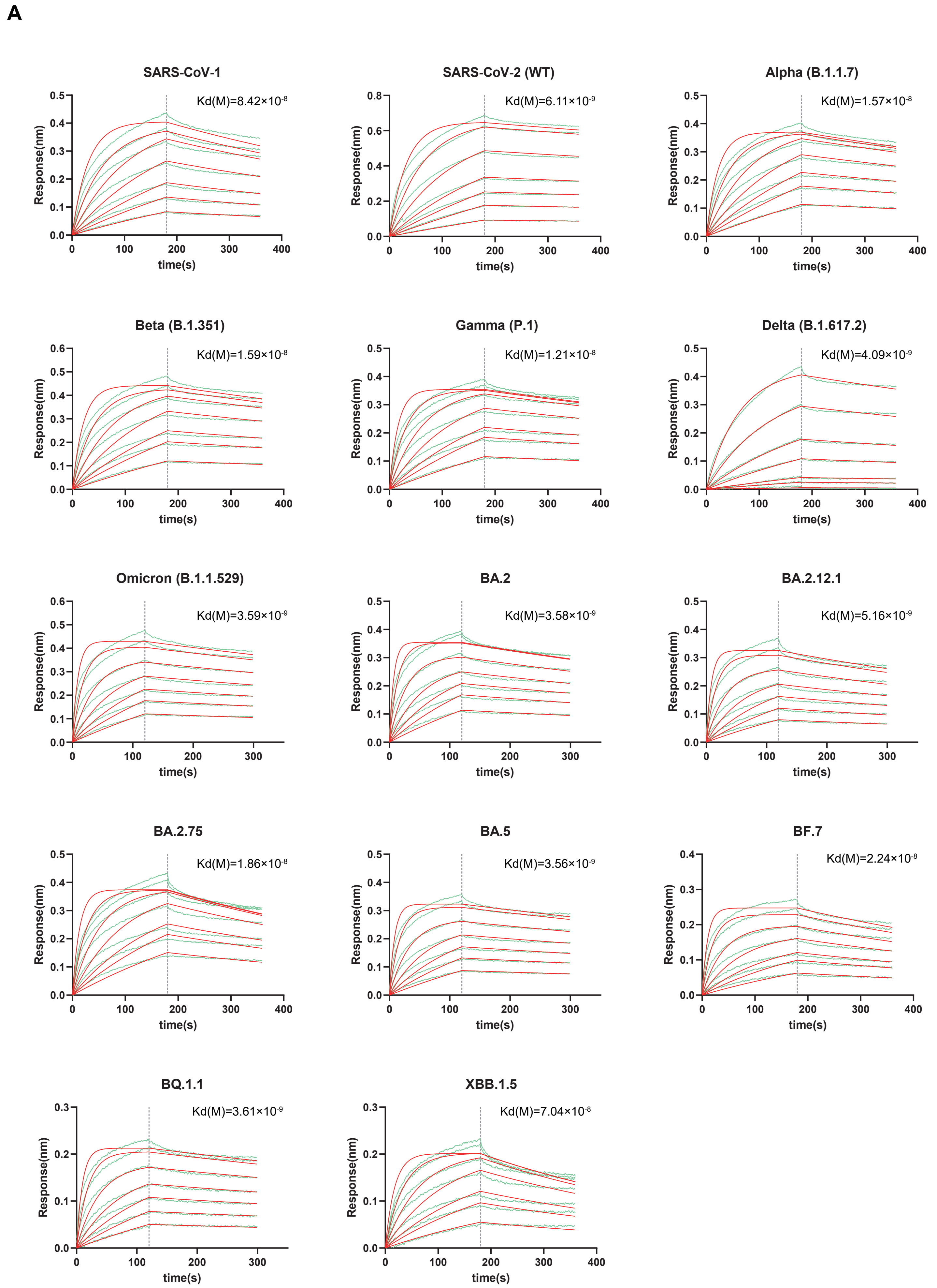

Fig. S5: Affinity of B11-E8-F3 with 14 RBD proteins.

Figure S6

A

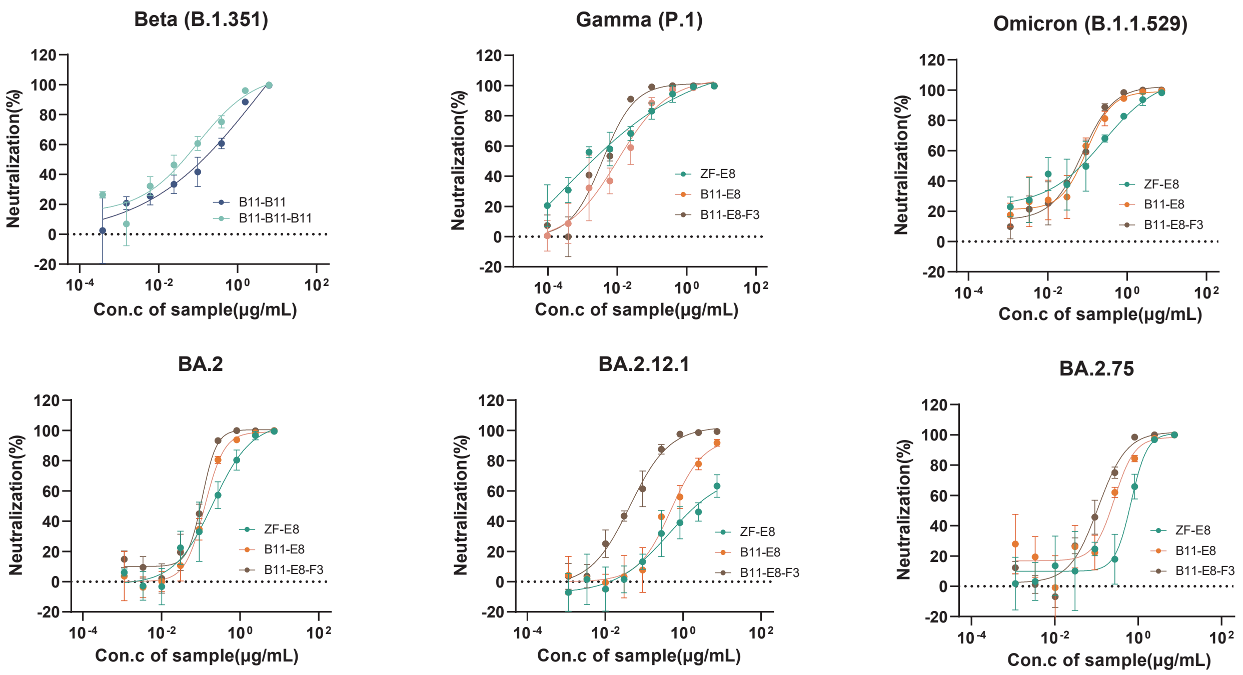

Beta (B.1.351)
Gamma (P.1)
Omicron (B.1.1.529)
BA.2
BA.2.12.1
BA.2.75
Neutralization(%)
Con.c of sample(μg/mL)
B11-B11
B11-B11-B11
ZF-E8
B11-E8
B11-E8-F3

B

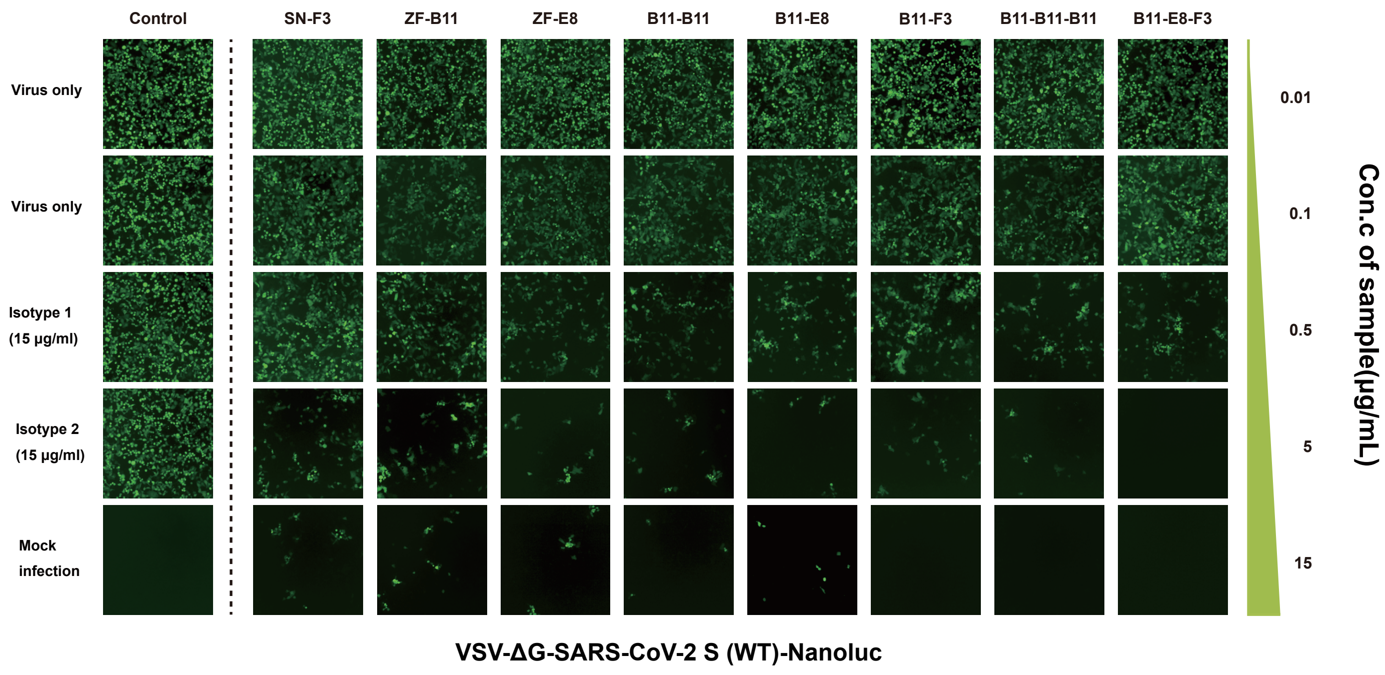

Control
SN-F3
ZF-B11
ZF-E8
B11-B11
B11-E8
B11-F3
B11-B11-B11
B11-E8-F3
Virus only
Virus only
Isotype 1
(15 μg/ml)
Isotype 2
(15 μg/ml)
Mock
infection
0.01
0.1
0.5
5
15
Con.c of sample(μg/mL)
VSV-ΔG-SARS-CoV-2 S (WT)-Nanoluc

C

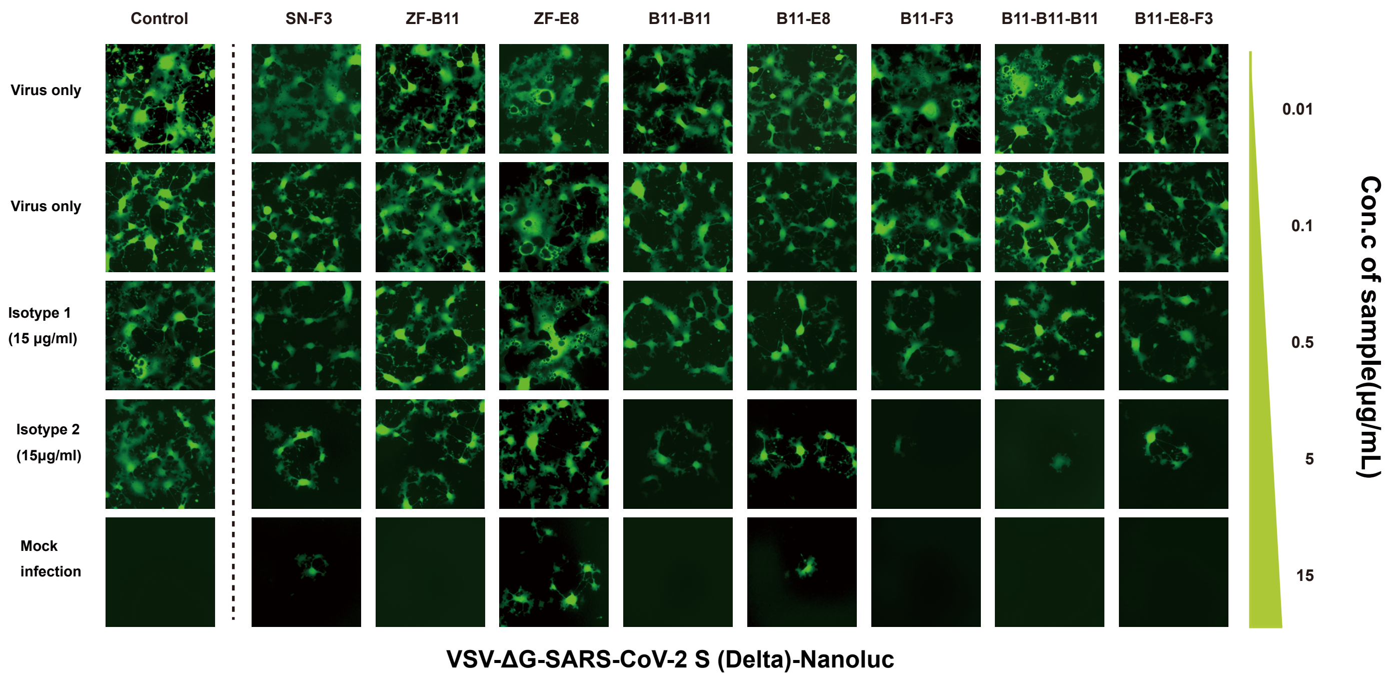

D

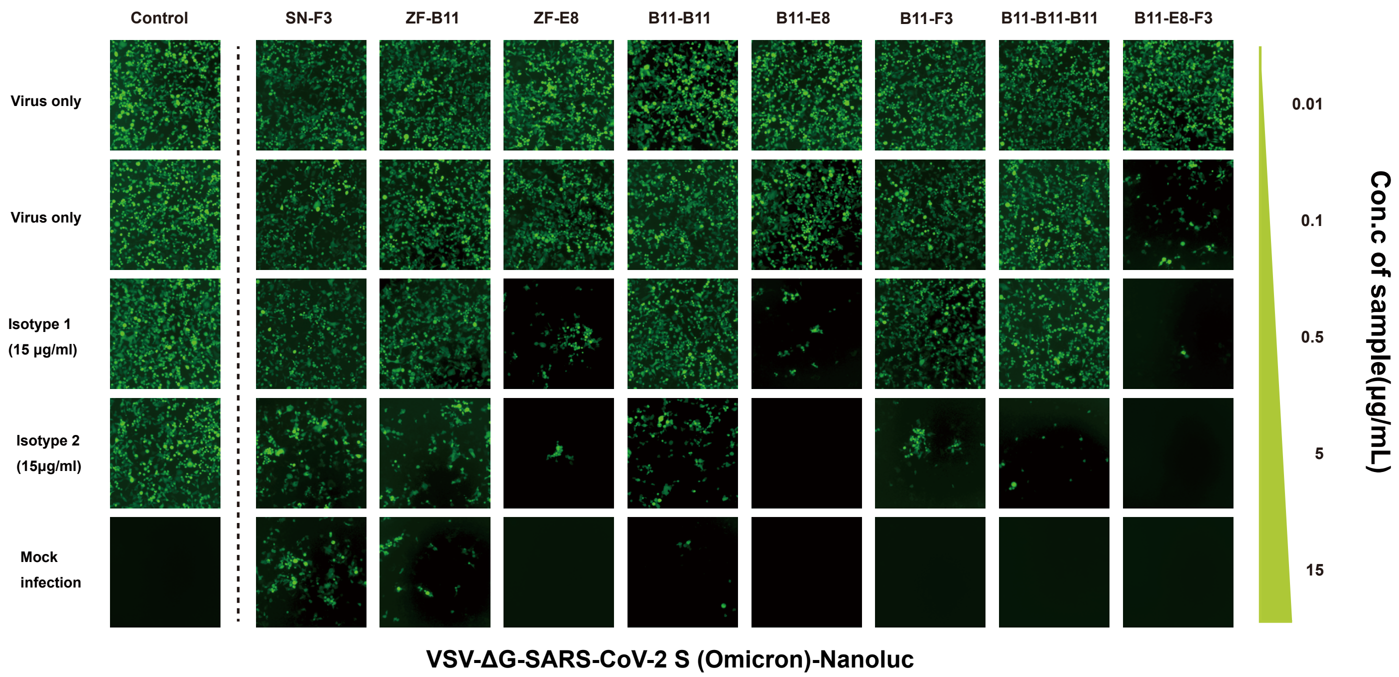

Fig. S6: Neutralizing activity of designed nanobodies against pseudoviruses.
(A) Neutralizing activity of highly effective neutralizing activity nanobodies against HIV pseudoviruses of SARS-CoV-2 VOCs (n=3).
(B-D) Neutralizing activity assay of multivalent nanobodies against VSV-GFP pesudoviruses (WT/Delta/Omicron) (200×).

Table S1

Immunization schedule

Days	Immunization steps	Antigen (Omicron) dose	Adjuvant	Immune site	Note
-4	blood collection	-	-	jugular vein	4-5 mL
0	1st	350μg	Freund's complete adjuvant	Subcutaneous (neck)	
7	2nd	350μg	Freund's complete adjuvant	Subcutaneous (neck)	
14	3rd	350μg	Freund's incomplete adjuvant	Subcutaneous (neck)	
21	4th	350μg	Freund's incomplete adjuvant	Subcutaneous (neck)	
28	5th	350μg	Freund's incomplete adjuvant	Subcutaneous (neck)	4-5 mL blood was collected for titer detection
35	6th	350μg	Freund's incomplete adjuvant	Subcutaneous (neck)	The adjuvant and antigen are not mixed, and are injected subcutaneously at a distance of 5 cm

Table S2

Coronavirus RBD/S protein	The EC50 binding activity (nM)				Coronavirus pseudovirus	The IC50 neutralizing activity (nM)			
	SN-F3	ZF-B11	ZF-E8	S309		SN-F3	ZF-B11	ZF-E8	S309
SARS-CoV-1	3.62	1.58	-	0.20	SARS-CoV-1	20.24	>100	-	24.39
SARS-CoV-2 (WT)	2.53	1.34	0.37	0.63	SARS-CoV-2 (WT)	2.18	0.75	0.18	4.98
Alpha (B.1.1.7)	7.34	2.20	0.59	2.68	Alpha (B.1.1.7)	11.71	0.81	0.20	2.92
Beta (B.1.351)	4.96	1.46	0.28	1.51	Beta (B.1.351)	17.43	12.41	0.04	2.11
Gamma (P.1)	5.27	1.56	0.24	1.25	Gamma (P.1)	5.13	1.64	0.03	13.51
Delta (B.1.617.2)	1.71	1.01	-	0.34	Delta (B.1.617.2)	22.53	14.71	-	3.53
Omicron (B.1.1.529)	>10	0.89	0.21	-	Omicron (B.1.1.529)	>100	44.29	3.466	-
BA.2	>10	1.11	0.29	-	BA.2	>100	63.7	0.76	>100
BA.2.12.1	>10	1.76	>1	-	BA.2.12.1	>100	30.38	14.49	>100
BA.2.75	>10	1.75	0.61	>10	BA.2.75	>100	>100	1.93	81.87
BA.5	3.49	0.45	-	-	BA.5	>100	41.37	-	-
BF.7	>10	1.22	-	-	BF.7	>100	78.97	-	-
BQ.1.1	2.23	0.66	-	-	BQ.1.1	>100	82.18	-	-
XBB.1.5	>10	0.22	>1	0.59	XBB.1.5	-	>100	33.93	>100

Note："-" means not binding.

Table S3

Antibody	Live virus	Concentration of Antibody(nM)						
		2000	500	125	31.25	7.81	1.95	0.49
IgG Isotype	SARS-CoV-2 (WT)	++++	++++	++++	++++	++++	++++	++++
		++++	++++	++++	++++	++++	++++	++++
		++++	++++	++++	++++	++++	++++	++++
	Delta	++++	++++	++++	++++	++++	++++	++++
		++++	++++	++++	++++	++++	++++	++++
		++++	++++	++++	++++	++++	++++	++++
	BA.2	+++	++++	++++	++++	++++	++++	++++
		+++	++++	++++	++++	++++	++++	++++
		+++	++++	++++	++++	++++	++++	++++
S309	SARS-CoV-2 (WT)	-	-	++++	++++	++++	++++	++++
		-	-	++++	++++	++++	++++	++++
		-	-	++	++++	++++	++++	++++
	Delta	-	-	-	-	+	++++	++++
		-	-	-	-	++	++++	++++
		-	-	-	+++	+++	++++	++++
	BA.2	+++	+++	+++	+++	+++	++++	++++
		+++	+++	+++	+++	+++	++++	++++
		+++	+++	+++	+++	+++	++++	++++
SN-F3	SARS-CoV-2 (WT)	-	-	-	++++	++++	++++	++++
		-	-	++++	++++	++++	++++	++++
		-	-	++++	++++	++++	++++	++++
	Delta	-	-	-	++++	++++	++++	++++
		-	-	-	++++	++++	++++	++++
		-	-	+++	++++	++++	++++	++++
	BA.2	-	++++	++++	++++	++++	++++	++++
		-	++++	++++	++++	++++	++++	++++
		-	++++	++++	++++	++++	++++	++++
ZF-B11	SARS-CoV-2 (WT)	++++	++++	++++	++++	++++	++++	++++
		+++	++++	++++	++++	++++	++++	++++
		+++	++++	++++	++++	++++	++++	++++
	Delta	-	-	-	++++	++++	++++	++++
		-	-	-	++++	++++	++++	++++
		-	-	-	++++	++++	++++	++++
	BA.2	-	-	++	++++	++++	++++	++++
		-	-	++	++++	++++	++++	++++

		-	-	+++	++++	++++	++++	++++
ZF-E8	SARS-CoV-2 (WT)	-	-	-	++++	++++	++++	++++
		-	-	-	++++	++++	++++	++++
		-	-	-	++++	++++	++++	++++
	Delta	++++	++++	++++	++++	++++	++++	++++
		++++	++++	++++	++++	++++	++++	++++
		++++	++++	++++	++++	++++	++++	++++
	Omicron	-	-	-	-	-	-	+
		-	-	-	-	-	-	+
		-	-	-	-	-	++	++++

Note: "++++" means severe, "+++" means mild, "++" means light, "+" means subtle, "-" means negative.

Table S4

SN-F3 & RBD

VHH	RBD	Interaction	VHH	RBD	Interaction
Y53	Y505	Pi-Pi Stacking	D113	T376	Hydrogen Bonds
T60	R403	Hydrogen Bonds		K378	Hydrogen Bonds
S103	N501	Hydrogen Bonds	S117	K378	Hydrogen Bonds
G105	N501	Hydrogen Bonds		R408	Hydrogen Bonds
T106	N439	Hydrogen Bonds	H119	Y508	Hydrogen Bonds
	Q498	Hydrogen Bonds	R120	N422	Hydrogen Bonds
	P499	Hydrogen Bonds	Y121	E406	Hydrogen Bonds
Y107	G502	Hydrogen Bonds		Y495	Weak H-Bonds
	Y505	Hydrogen Bonds		G504	Weak H-Bonds
Y108	Q506	Hydrogen Bonds	G123	G404	Hydrogen Bonds
W109	S438	Hydrogen Bonds	D130	V503	Hydrogen Bonds
S112	T376	Hydrogen Bonds			
	A435	Hydrogen Bonds			

ZF-E8 & RBD

VHH	RBD	Interaction
D30	A372	Hydrogen Bonds
E31	S373	Polar Bonds
K53	N440	Hydrogen Bonds
	V503	Hydrogen Bonds
D54	N439	Hydrogen Bonds
	Q506	Hydrogen Bonds

ZF-B11 & RBD

VHH	RBD	Interaction
D33	N370	Hydrogen Bonds
R53	Y369	Hydrogen Bonds
	N370	Hydrogen Bonds
S56	T385	Polar Bonds

Note: Mutations at nanobodies' recognization sites during evolution were marked with red. Amino acids in different domains were highlighted with colors (orange: Domain I, purple: Domain II, green: Domain III).

Table S5

Coronavirus pseudovirus	The IC50 neutralizing activity (μg/mL)								
	SN-F3	ZF-B11	ZF-E8	B11-B11	B11-E8	B11-F3	B11-B11-B11	B11-F3-B11	B11-E8-F3
SARS-CoV-1	0.8847	20.05	-	12.06	6461	1.541	3.835	0.5659	0.8065
SARS-CoV-2 (WT)	2.355	1.792	0.08405	1.002	0.2002	0.9763	0.2854	0.3966	0.1041
Alpha (B.1.1.7)	3.437	0.06859	0.5512	1.727	0.4382	1.65	2.068	1.423	0.1533
Beta (B.1.351)	2.862	0.9343	0.24	0.9818	0.2653	1.255	0.6048	0.5799	0.0759
Gamma (P.1)	1.781	1.36	0.2404	0.6008	0.01034	1.064	0.5524	0.5064	0.003767
Delta (B.1.617.2)	2.961	0.8954	-	1.562	9.575	1.901	0.7469	1.518	1.905
Omicron (B.1.529)	-	2.155	0.2564	1.346	0.09599	2.147	0.5289	0.7172	0.07531
BA.2	78.43	5.966	0.2031	2.575	0.1254	3.911	2.02	2.756	0.1083
BA.2.12.1	64.57	8.08	0.4357	3.988	0.4841	4.626	1.566	3.309	0.04291
BA.2.75	>100	31.74	0.6759	8.605	0.2686	10.56	3.159	8.902	0.11
BA.5	>100	2.853	-	1.236	9.204	2.19	0.9358	1.879	0.7077
BF.7	>100	8.716	-	6.454	79.4	7.641	5.055	3.291	5.058
BQ.1.1	>100	9.853	-	7.837	70.5	8.082	7.202	8.942	9.46
XBB.1.5	-	5.327	0.6119	4.027	1.146	5.688	2.263	4.84	0.0399
EG.5	>100	22.05	4.943	7.749	19.46	9.057	6.571	8.014	0.1533

Note: “-” means not binding.

Table S6

REAGENT OR RESOURCE	SOURCE	IDENTIFIER
**Bacterial and virus strains**		
TG1 Electrocompetent Cells	LGC Biosearch Technologies	Cat# 60502-2
M13KO7 Helper Phage	NEB	Cat# N0315S
SARS-CoV-2（C57M14）[62]、SARS-CoV-2（WH-01、Delta、BA.2、BA.5、XBB.1.16）	Infected donors	N/A
**Chemicals, peptides and recombinant proteins**		
SMM 293-TII Expression Medium	Sino Biological	Cat# M293TII-1L
Sinofection Transfection Reagent	Sino Biological	Cat# STF02-5ML
SMS 293-SUPI cell culture supplement	Sino Biological	Cat# M293-SUPI-100mL
ACE2-Bio Protein	Sino Biological	Cat# 10108-H08H-B
SARS-CoV-1 Spike S1+S2 Protein	Sino Biological	Cat# 40634-V08B
SARS-CoV-2 (WT) Spike RBD Protein	Sino Biological	Cat# 40592-V08B
Alpha (B.1.1.7) Spike S1+S2 Protein	Sino Biological	Cat# 40589-V08B6
Beta (B.1.351) Spike S1+S2 Protein	Sino Biological	Cat# 40589-V08B9
Gamma (P.1) Spike S1+S2 Protein	Sino Biological	Cat# 40589-V08B10
Delta (B.1.617.2) Spike RBD Protein	Sino Biological	Cat# 40592-V08H90
Omicron (B.1.1.529) Spike RBD Protein	Sino Biological	Cat# 40592-V08H121
Omicron (BA.2) Spike RBD Protein	Sino Biological	Cat# 40592-V08H123
Omicron (BA.2.75) Spike RBD Protein	Sino Biological	Cat# 40592-V08H135
Omicron (BA.4/BA.5) Spike RBD Protein	Sino Biological	Cat# 40592-V08H130
Omicron (BA.2.12.1) Spike RBD Protein	Sino Biological	Cat# 40592-V08H132
Omicron (BF.7) Spike RBD Protein	Sino Biological	Cat# 40592-V08H140
Omicron (BQ.1.1) Spike RBD Protein	Sino Biological	Cat# 40592-V08H143
Omicron (XBB.1.5) Spike RBD Protein	Sino Biological	Cat# 40592-V08H146
Omicron (XBB.1.5) Spike S1+S2 trimer Protein	Sino Biological	Cat# 40589-V08H45
**Antibodies**		

Anti-M13-HRP	Sino Biological	Cat# 11973-MM05T-H
Rabbit Anti-Camel IgG（HRP）	Solarbio	Cat# SA283-HRP
Goat anti-human IgG H+L(HRP)	SeraCare	Cat# 5210-0159
Strepavdin-HRP	ThermoFisher	S911
S309	Dora Pinto et.al[51]	N/A
LXY08 IgG	prepared by laboratory	N/A
F61	Sinopharm Group	N/A
SA58	Sinovac	N/A
**Critical commercial assays**		
PBMC isolation kit	Tianjin Haoyang	Cat# LTS10771
RNA Easy Fast Tissue/Cell Kit	TIANGEN	Cat# DP451
Fast King RT Kit（With gDNase）	TIANGNE	Cat# KR116-01
Biotin Labeling Kit	Elabscience®	Cat# E-LK-B002
Luciferase Assay Kit	Beyotime	Cat# RG052M
The Nano-Glo® Luciferase Assay System	Promega	Cat# N1110
**Recombinant DNA**		
pComb3XTT vector	Addgene	Cat# 63891
pSG3$^{\Delta env}$-cmvFluc	Gift from China Academy of Food and Drug Control	
**Cell lines**		
HEK293T cells	ATCC	N/A
HEK293F cells	Sino Biological	N/A
ACE2-OE(HEK293T) cell	Sino Biological	N/A
Vero E6 cells	iCell Bioscience	N/A
CHO-S cell	Sino Biological	N/A
**Experimental models: Organisoms/strains**		
Golden Syrian Hamster	Beijing Vitalstar Biotechnology Co.,Ltd.	
BALB/c mice	Beijing Vitalstar Biotechnology Co.,Ltd.	
CAG-hACE2-IRES-Luc-Tg transgenic mice	Shanghai Model Organisms Center	
**Software and algorithms**		

Molecular simulation software	Insight II 2000
Data processing software	Graphpad Prism 9.4.0
Statistical analysis software	IBM SPSS Statistics 27
Image processing software	Adobe Illustrator 2024

Table S7

Viruses	S protein sequences
**SARS-CoV-1**	MFIFLLFLTLTSGSDLDRCTTFDDVQAPNYTQHTSSMRGVYYPDEIFRSDTLYLTQDLFLPFYS<br>NVTGFHTINHTFGNPVIPFKDGIYFAATEKSNVVRGWVFGSTMNNKSQSVIIINNSTNVVIRAC<br>NFELCDNPFFAVSKPMGTQTHTMIFDNAFNCTFEYISDAFSLDVSEKSGNFKHLREFVFKNKD<br>GFLYVYKGYQPIDVVRDLPSGFNTLKPIFKLPLGINITNFRAILTAFSPAQDIWGTSAAAYFVG<br>YLKPTTFMLKYDENGTITDAVDCSQNPLAELKCSVKSFEIDKGIYQTSNFRVVPSGDVVRFPN<br>ITNLCPFGEVFNATKFPSVYAWERKKISNCVADYSVLYNSTFFSTFKCYGVSATKLNDLCFSN<br>VYADSFVVKGDDVRQIAPGQTGVIADYNYKLPDDFMGCVLAWNTRNIDATSTGNYNYKYR<br>YLRHGKLRPFERDISNVPFSPDGKPCTPPALNCYWPLNDYGFYTTTGIGYQPYRVVVLSFELL<br>NAPATVCGPKLSTDLIKNQCVNFNFNGLTGTGVLTPSSKRFQPFQQFGRDVSDFTDSVRDPKT<br>SEILDISPCSFGGVSVITPGTNASSEVAVLYQDVNCTDVSTAIHADQLTPAWRIYSTGNNVFQT<br>QAGCLIGAEHVDTSYECDIPIGAGICASYHTVSLLRSTSQKSIVAYTMSLGADSSIAYSNNTIAI<br>PTNFSISITTEVMPVSMAKTSVDCNMYICGDSTECANLLLQYGSFCTQLNRALSGIAAEQDRN<br>TREVFAQVKQMYKTPTLKYFGGFNFSQILPDPLKPTKRSFIEDLLFNKVTLADAGFMKQYGE<br>CLGDINARDLICAQKFNGLTVLPPLLTDDMIAAYTAALVSGTATAGWTFGAGAALQIPFAMQ<br>MAYRFNGIGVTQNVLYENQKQIANQFNKAISQIQESLTTTSTALGKLQDVVNQNAQALNTLV<br>KQLSSNFGAISSVLNDILSRLDKVEAEVQIDRLITGRLQSLQTYVTQQLIRAAEIRASANLAAT<br>KMSECVLGQSKRVDFCGKGYHLMSFPQAAPHGVVFLHVTYVPSQERNFTTAPAICHEGKAY<br>FPREGVFVFNGTSWFITQRNFFSPQIITTDNTFVSGNCDVVIGIINNTVYDPLQPELDSFKEELD<br>KYFKNHTSPDVDLGDISGINASVVNIQKEIDRLNEVAKNLNESLIDLQELGKYEQYIKWPWY<br>VWLGFIAGLIAIVMVTILLCCMTSCCSCLKGACSCGSCC
**SARS-CoV-2 (WT)**	MFVFLVLLPLVSSQCVNLTTRTQLPPAYTNSFTRGVYYPDKVFRSSVLHSTQDLFLPFFSNVT<br>WFHAIHVSGTNGTKRFDNPVLPFNDGVYFASTEKSNIIRGWIFGTTLDSKTQSLLIVNNATNV<br>VIKVCEFQFCNDPFLGVYYHKNNKSWMESEFRVYSSANNCTFEYVSQPFLMDLEGKQGNFK<br>NLREFVFKNIDGYFKIYSKHTPINLVRDLPQGFSALEPLVDLPIGINITRFQTLLALHRSYLTPG<br>DSSSGWTAGAAAYYVGYLQPRTFLLKYNENGTITDAVDCALDPLSETKCTLKSFTVEKGIYQ<br>TSNFRVQPTESIVRFPNITNLCPFGEVFNATRFASVYAWNRKRISNCVADYSVLYNSASFSTFK<br>CYGVSPTKLNDLCFTNVYADSFVIRGDEVRQIAPGQTGKIADYNYKLPDDFTGCVIAWNSNN<br>LDSKVGGNYNYLYRLFRKSNLKPFERDISTEIYQAGSTPCNGVEGFNCYFPLQSYGFQPTNG<br>VGYQPYRVVVLSFELLHAPATVCGPKKSTNLVKNKCVNFNFNGLTGTGVLTESNKKFLPFQQ<br>FGRDIADTTDAVRDPQTLEILDITPCSFGGVSVITPGTNTSNQVAVLYQDVNCTEVPVAIHADQ<br>LTPTWRVYSTGSNVFQTRAGCLIGAEHVNNSYECDIPIGAGICASYQTQTNSPRRARSVASQSI<br>IAYTMSLGAENSVAYSNNSIAIPTNFTISVTTEILPVSMTKTSVDCTMYICGDSTECSNLLLQYG<br>SFCTQLNRALTGIAVEQDKNTQEVFAQVKQIYKTPPIKDFGGFNFSQILPDPSKPSKRSFIEDLL<br>FNKVTLADAGFIKQYGDCLGDIAARDLICAQKFNGLTVLPPLLTDEMIAQYTSALLAGTITSG<br>WTFGAGAALQIPFAMQMAYRFNGIGVTQNVLYENQKLIANQFNSAIGKIQDSLSSTASALGK<br>LQDVVNQNAQALNTLVKQLSSNFGAISSVLNDILSRLDKVEAEVQIDRLITGRLQSLQTYVTQ<br>QLIRAAEIRASANLAATKMSECVLGQSKRVDFCGKGYHLMSFPQSAPHGVVFLHVTYVPAQ<br>EKNFTTAPAICHDGKAHFPREGVFVSNGTHWFVTQRNFYEPQIITTDNTFVSGNCDVVIGIVN<br>NTVYDPLQPELDSFKEELDKYFKNHTSPDVDLGDISGINASVVNIQKEIDRLNEVAKNLNESLI<br>DLQELGKYEQYIKWPWYIWLGFIAGLIAIVMVTIMLCCMTSCCSCLKGCCSCGSCC
**Alpha**	MFVFLVLLPLVSSQCVNLTTRTQLPPAYTNSFTRGVYYPDKVFRSSVLHSTQDLFLPFFSNVT

**(B.1.1.7)**	WFHAISGTNGTKRFDNPVLPFNDGVYFASTEKSNIIRGWIFGTTLDSKTQSLLIVNNATNVVIK<br>VCEFQFCNDPFLGVYHKNNKSWMESEFRVYSSANNCTFEYVSQPFLMDLEGKQGNFKNLRE<br>FVFKNIDGYFKIYSKHTPINLVRDLPQGFSALEPLVDLPIGINITRFQTLLALHRSYLTPGDSSSG<br>WTAGAAAYYVGYLQPRTFLLKYNENGTITDAVDCALDPLSETKCTLKSFTVEKGIYQTSNFR<br>VQPTESIVRFPNITNLCPFGEVFNATRFASVYAWNRKRISNCVADYSVLYNSASFSTFKCYGVS<br>PTKLNDLCFTNVYADSFVIRGDEVRQIAPGQTGKIADYNYKLPDDFTGCVIAWNSNNLDSKV<br>GGNYNYLYRLFRKSNLKPFERDISTEIYQAGSTPCNGVEGFNCYFPLQSYGFQPTYGVGYQP<br>YRVVVLSFELLHAPATVCGPKKSTNLVKNKCVNFNFNGLTGTGVLTESNKKFLPFQQFGRDID<br>DTTDAVRDPQTLEILDITPCSFGGVSVITPGTNTSNQVAVLYQGVNCTEVPVAIHADQLTPTWR<br>VYSTGSNVFQTRAGCLIGAEHVNNSYECDIPIGAGICASYQTQTNSHRRARSVASQSIIAYTMS<br>LGAENSVAYSNNSIAIPINFTISVTTEILPVSMTKTSVDCTMYICGDSTECSNLLLQYGSFCTQL<br>NRALTGIAVEQDKNTQEVFAQVKQIYKTPPIKDFGGFNFSQILPDPSKPSKRSFIEDLLFNKVTL<br>ADAGFIKQYGDCLGDIAARDLICAQKFNGLTVLPPLLTDEMIAQYTSALLAGTITSGWTFGAG<br>AALQIPFAMQMAYRFNGIGVTQNVLYENQKLIANQFNSAIGKIQDSLSSTASALGKLQDVVN<br>QNAQALNTLVKQLSSNFGAISSVLNDILARLDKVEAEVQIDRLITGRLQSLQTYVTQQLIRAA<br>EIRASANLAATKMSECVLGQSKRVDFCGKGYHLMSFPQSAPHGVVFLHVTYVPAQEKNFTT<br>APAICHDGKAHFPREGVFVSNGTHWFVTQRNFYEPQIITTHNTFVSGNCDVVIGIVNNTVYDP<br>LQPELDSFKEELDKYFKNHTSPDVDLGDISGINASVVNIQKEIDRLNEVAKNLNESLIDLQELG<br>KYEQYIKWPWYIWLGFIAGLIAIVMVTIMLCCMTSCCSCLKGCCSCGSCC
**Beta (B.1.351)**	MFVFLVLLPLVSSQCVNFTTRTQLPPAYTNSFTRGVYYPDKVFRSSVLHSTQDLFLPFFSNVT<br>WFHAIHVSGTNGTKRFANPVLPFNDGVYFASTEKSNIIRGWIFGTTLDSKTQSLLIVNNATNV<br>VIKVCEFQFCNDPFLGVYYHKNNKSWMESEFRVYSSANNCTFEYVSQPFLMDLEGKQGNFK<br>NLREFVFKNIDGYFKIYSKHTPINLVRGLPQGFSALEPLVDLPIGINITRFQTLLALHRSYLTPG<br>DSSSGWTAGAAAYYVGYLQPRTFLLKYNENGTITDAVDCALDPLSETKCTLKSFTVEKGIYQ<br>TSNFRVQPTESIVRFPNITNLCPFGEVFNATRFASVYAWNRKRISNCVADYSVLYNSASFSTFK<br>CYGVSPTKLNDLCFTNVYADSFVIRGDEVRQIAPGQTGNIADYNYKLPDDFTGCVIAWNSNN<br>LDSKVGGNYNYLYRLFRKSNLKPFERDISTEIYQAGSTPCNGVKGFNCYFPLQSYGFQPTYG<br>VGYQPYRVVVLSFELLHAPATVCGPKKSTNLVKNKCVNFNFNGLTGTGVLTESNKKFLPFQQ<br>FGRDIADTTDAVRDPQTLEILDITPCSFGGVSVITPGTNTSNQVAVLYQGVNCTEVPVAIHADQ<br>LTPTWRVYSTGSNVFQTRAGCLIGAEHVNNSYECDIPIGAGICASYQTQTNSPRRARSVASQSI<br>IAYTMSLGVENSVAYSNNSIAIPTNFTISVTTEILPVSMTKTSVDCTMYICGDSTECSNLLLQYG<br>SFCTQLNRALTGIAVEQDKNTQEVFAQVKQIYKTPPIKDFGGFNFSQILPDPSKPSKRSFIEDLL<br>FNKVTLADAGFIKQYGDCLGDIAARDLICAQKFNGLTVLPPLLTDEMIAQYTSALLAGTITSG<br>WTFGAGAALQIPFAMQMAYRFNGIGVTQNVLYENQKLIANQFNSAIGKIQDSLSSTASALGK<br>LQDVVNQNAQALNTLVKQLSSNFGAISSVLNDILSRLDKVEAEVQIDRLITGRLQSLQTYVTQ<br>QLIRAAEIRASANLAATKMSECVLGQSKRVDFCGKGYHLMSFPQSAPHGVVFLHVTYVPAQ<br>EKNFTTAPAICHDGKAHFPREGVFVSNGTHWFVTQRNFYEPQIITTDNTFVSGNCDVVIGIVN<br>NTVYDPLQPELDSFKEELDKYFKNHTSPDVDLGDISGINASVVNIQKEIDRLNEVAKNLNESLI<br>DLQELGKYEQYIKWPWYIWLGFIAGLIAIVMVTIMLCCMTSCCSCLKGCCSCGSCC
**Gamma (P.1)**	MFVFLVLLPLVSSQCVNFTNRTQLPSAYTNSFTRGVYYPDKVFRSSVLHSTQDLFLPFFSNVT<br>WFHAIHVSGTNGTKRFDNPVLPFNDGVYFASTEKSNIIRGWIFGTTLDSKTQSLLIVNNATNV<br>VIKVCEFQFCNYPFLGVYYHKNNKSWMESEFRVYSSANNCTFEYVSQPFLMDLEGKQGNFK<br>NLSEFVFKNIDGYFKIYSKHTPINLVRDLPQGFSALEPLVDLPIGINITRFQTLLALHRSYLTPG<br>DSSSGWTAGAAAYYVGYLQPRTFLLKYNENGTITDAVDCALDPLSETKCTLKSFTVEKGIYQ

	TSNFRVQPTESIVRFPNITNLCPFGEVFNATRFASVYAWNRKRISNCVADYSVLYNSASFSTFK<br>CYGVSPTKLNDLCFTNVYADSFVIRGDEVRQIAPGQTGTIADYNYKLPDDFTGCVIAWNSNN<br>LDSKVGGNYNYLYRLFRKSNLKPFERDISTEIYQAGSTPCNGVKGFNCYFPLQSYGFQPTYG<br>VGYQPYRVVVLSFELLHAPATVCGPKKSTNLVKNKCVNFNFNGLTGTGVLTESNKKFLPFQQ<br>FGRDIADTTDAVRDPQTLEILDITPCSFGGVSVITPGTNTSNQVAVLYQGVNCTEVPVAIHADQ<br>LTPTWRVYSTGSNVFQTRAGCLIGAEYVNNSYECDIPIGAGICASYQTQTNSPRRARSVASQSI<br>IAYTMSLGAENSVAYSNNSIAIPTNFTISVTTEILPVSMTKTSVDCTMYICGDSTECSNLLLQYG<br>SFCTQLNRALTGIAVEQDKNTQEVFAQVKQIYKTPPIKDFGGFNFSQILPDPSKPSKRSFIEDLL<br>FNKVTLADAGFIKQYGDCLGDIAARDLICAQKFNGLTVLPPLLTDEMIAQYTSALLAGTITSG<br>WTFGAGAALQIPFAMQMAYRFNGIGVTQNVLYENQKLIANQFNSAIGKIQDSLSSTASALGK<br>LQDVVNQNAQALNTLVKQLSSNFGAISSVLNDILSRLDKVEAEVQIDRLITGRLQSLQTYVTQ<br>QLIRAAEIRASANLAAIKMSECVLGQSKRVDFCGKGYHLMSFPQSAPHGVVFLHVTYVPAQE<br>KNFTTAPAICHDGKAHFPREGVFVSNGTHWFVTQRNFYEPQIITTDNTFVSGNCDVVIGIVNN<br>TVYDPLQPELDSFKEELDKYFKNHTSPDVDLGDISGINASFVNIQKEIDRLNEVAKNLNESLID<br>LQELGKYEQYIKWPWYIWLGFIAGLIAIVMVTIMLCCMTSCCSCLKGCCSCGSCC
**Delta (B.1.617.2)**	MFVFLVLLPLVSSQCVNLRTRTQLPPAYTNSFTRGVYYPDKVFRSSVLHSTQDLFLPFFSNVT<br>WFHAIHVSGTNGTKRFDNPVLPFNDGVYFASTEKSNIIRGWIFGTTLDSKTQSLLIVNNATNV<br>VIKVCEFQFCNDPFLDVYYHKNNKSWMESEVYSSANNCTFEYVSQPFLMDLEGKQGNFKN<br>LREFVFKNIDGYFKIYSKHTPINLVRDLPQGFSALEPLVDLPIGINITRFQTLLALHRSYLTPGDS<br>SSGWTAGAAAYYVGYLQPRTFLLKYNENGTITDAVDCALDPLSETKCTLKSFTVEKGIYQTS<br>NFRVQPTESIVRFPNITNLCPFGEVFNATRFASVYAWNRKRISNCVADYSVLYNSASFSTFKCY<br>GVSPTKLNDLCFTNVYADSFVIRGDEVRQIAPGQTGKIADYNYKLPDDFTGCVIAWNSNNLD<br>SKVGGNYNYRYRLFRKSNLKPFERDISTEIYQAGSKPCNGVEGFNCYFPLQSYGFQPTNGVG<br>YQPYRVVVLSFELLHAPATVCGPKKSTNLVKNKCVNFNFNGLTGTGVLTESNKKFLPFQQFG<br>RDIADTTDAVRDPQTLEILDITPCSFGGVSVITPGTNTSNQVAVLYQGVNCTEVPVAIHADQLT<br>PTWRVYSTGSNVFQTRAGCLIGAEHVNNSYECDIPIGAGICASYQTQTNSRRRARSVASQSIIA<br>YTMSLGAENSVAYSNNSIAIPTNFTISVTTEILPVSMTKTSVDCTMYICGDSTECSNLLLQYGS<br>FCTQLNRALTGIAVEQDKNTQEVFAQVKQIYKTPPIKDFGGFNFSQILPDPSKPSKRSFIEDLLF<br>NKVTLADAGFIKQYGDCLGDIAARDLICAQKFNGLTVLPPLLTDEMIAQYTSALLAGTITSG<br>WTFGAGAALQIPFAMQMAYRFNGIGVTQNVLYENQKLIANQFNSAIGKIQDSLSSTASALGK<br>LQNVVNQNAQALNTLVKQLSSNFGAISSVLNDILSRLDKVEAEVQIDRLITGRLQSLQTYVTQ<br>QLIRAAEIRASANLAATKMSECVLGQSKRVDFCGKGYHLMSFPQSAPHGVVFLHVTYVPAQ<br>EKNFTTAPAICHDGKAHFPREGVFVSNGTHWFVTQRNFYEPQIITTDNTFVSGNCDVVIGIVN<br>NTVYDPLQPELDSFKEELDKYFKNHTSPDVDLGDISGINASVVNIQKEIDRLNEVAKNLNESLI<br>DLQELGKYEQYIKWPWYIWLGFIAGLIAIVMVTIMLCCMTSCCSCLKGCCSCGSCC
**Omicron (B.1.1.529)**	MFVFLVLLPLVSSQCVNLTTRTQLPPAYTNSFTRGVYYPDKVFRSSVLHSTQDLFLPFFSNVT<br>WFHVISGTNGTKRFDNPVLPFNDGVYFASIEKSNIIRGWIFGTTLDSKTQSLLIVNNATNVVIK<br>VCEFQFCNDPFLDHKNNKSWMESEFRVYSSANNCTFEYVSQPFLMDLEGKQGNFKNLREFV<br>FKNIDGYFKIYSKHTPIIVREPEDLPQGFSALEPLVDLPIGINITRFQTLLALHRSYLTPGDSSSG<br>WTAGAAAYYVGYLQPRTFLLKYNENGTITDAVDCALDPLSETKCTLKSFTVEKGIYQTSNFR<br>VQPTESIVRFPNITNLCPFDEVFNATRFASVYAWNRKRISNCVADYSVLYNLAPFFTFKCYGVS<br>PTKLNDLCFTNVYADSFVIRGDEVRQIAPGQTGNIADYNYKLPDDFTGCVIAWNSNKLDSKV<br>SGNYNYLYRLFRKSNLKPFERDISTEIYQAGNKPCNGVAGFNCYFPLRSYSFRPTYGVGHQPY<br>RVVVLSFELLHAPATVCGPKKSTNLVKNKCVNFNFNGLKGTGVLTESNKKFLPFQQFGRDIA

	DTTDAVRDPQTLEILDITPCSFGGVSVITPGTNTSNQVAVLYQGVNCTEVPVAIHADQLTPTWR<br>VYSTGSNVFQTRAGCLIGAEYVNNSYECDIPIGAGICASYQTQTKSHRRARSVASQSIIAYTMS<br>LGAENSVAYSNNSIAIPTNFTISVTTEILPVSMTKTSVDCTMYICGDSTECSNLLLQYGSFCTQL<br>KRALTGIAVEQDKNTQEVFAQVKQIYKTPPIKYFGGFNFSQILPDPSKPSKRSFIEDLLFNKVTL<br>ADAGFIKQYGDCLGDIAARDLICAQKFKGLTVLPPLLTDEMIAQYTSALLAGTITSGWTFGAG<br>AALQIPFAMQMAYRFNGIGVTQNVLYENQKLIANQFNSAIGKIQDSLSSTASALGKLQDVVN<br>HNAQALNTLVKQLSSKFGAISSVLNDIFSRLDKVEAEVQIDRLITGRLQSLQTYVTQQLIRAA<br>EIRASANLAATKMSECVLGQSKRVDFCGKGYHLMSFPQSAPHGVVFLHVTYVPAQEKNFTT<br>APAICHDGKAHFPREGVFVSNGTHWFVTQRNFYEPQIITTDNTFVSGNCDVVIGIVNNTVYDP<br>LQPELDSFKEELDKYFKNHTSPDVDLGDISGINASVVNIQKEIDRLNEVAKNLNESLIDLQELG<br>KYEQYIKWPWYIWLGFIAGLIAIVMVTIMLCCMTSCCSCLKGCCSCGSCC
**BA.2**	MFVFLVLLPLVSSQCVNLITRTQSYTNSFTRGVYYPDKVFRSSVLHSTQDLFLPFFSNVTWFH<br>AIHVSGTNGTKRFDNPVLPFNDGVYFASTEKSNIIRGWIFGTTLDSKTQSLLIVNNATNVVIKV<br>CEFQFCNDPFLDVYYHKNNKSWMESEFRVYSSANNCTFEYVSQPFLMDLEGKQGNFKNLRE<br>FVFKNIDGYFKIYSKHTPINLGRDLPQGFSALEPLVDLPIGINITRFQTLLALHRSYLTPGDSSSG<br>WTAGAAAYYVGYLQPRTFLLKYNENGTITDAVDCALDPLSETKCTLKSFTVEKGIYQTSNFR<br>VQPTESIVRFPNITNLCPFDEVFNATRFASVYAWNRKRISNCVADYSVLYNFAPFFAFKCYGVS<br>PTKLNDLCFTNVYADSFVIRGNEVSQIAPGQTGNIADYNYKLPDDFTGCVIAWNSNKLDSKV<br>GGNYNYLYRLFRKSNLKPFERDISTEIYQAGNKPCNGVAGFNCYFPLRSYGFRPTYGVGHQP<br>YRVVVLSFELLHAPATVCGPKKSTNLVKNKCVNFNFNGLTGTGVLTESNKKFLPFQQFGRDIA<br>DTTDAVRDPQTLEILDITPCSFGGVSVITPGTNTSNQVAVLYQGVNCTEVPVAIHADQLTPTWR<br>VYSTGSNVFQTRAGCLIGAEYVNNSYECDIPIGAGICASYQTQTKSHRRARSVASQSIIAYTMS<br>LGAENSVAYSNNSIAIPTNFTISVTTEILPVSMTKTSVDCTMYICGDSTECSNLLLQYGSFCTQL<br>KRALTGIAVEQDKNTQEVFAQVKQIYKTPPIKYFGGFNFSQILPDPSKPSKRSFIEDLLFNKVTL<br>ADAGFIKQYGDCLGDIAARDLICAQKFNGLTVLPPLLTDEMIAQYTSALLAGTITSGWTFGAG<br>AALQIPFAMQMAYRFNGIGVTQNVLYENQKLIANQFNSAIGKIQDSLSSTASALGKLQDVVN<br>HNAQALNTLVKQLSSKFGAISSVLNDILSRLDKVEAEVQIDRLITGRLQSLQTYVTQQLIRAA<br>EIRASANLAATKMSECVLGQSKRVDFCGKGYHLMSFPQSAPHGVVFLHVTYVPAQEKNFTT<br>APAICHDGKAHFPREGVFVSNGTHWFVTQRNFYEPQIITTDNTFVSGNCDVVIGIVNNTVYDP<br>LQPELDSFKEELDKYFKNHTSPDVDLGDISGINASVVNIQKEIDRLNEVAKNLNESLIDLQELG<br>KYEQYIKWPWYIWLGFIAGLIAIVMVTIMLCCMTSCCSCLKGCCSCGSCC
**BA.2.12.1**	MFVFLVLLPLVSSQCVNLITRTQSYTNSFTRGVYYPDKVFRSSVLHSTQDLFLPFFSNVTWFH<br>AIHVSGTNGTKRFDNPVLPFNDGVYFASTEKSNIIRGWIFGTTLDSKTQSLLIVNNATNVVIKV<br>CEFQFCNDPFLDVYYHKNNKSWMESEFRVYSSANNCTFEYVSQPFLMDLEGKQGNFKNLRE<br>FVFKNIDGYFKIYSKHTPINLGRDLPQGFSALEPLVDLPIGINITRFQTLLALHRSYLTPGDSSSG<br>WTAGAAAYYVGYLQPRTFLLKYNENGTITDAVDCALDPLSETKCTLKSFTVEKGIYQTSNFR<br>VQPTESIVRFPNITNLCPFDEVFNATRFASVYAWNRKRISNCVADYSVLYNFAPFFAFKCYGVS<br>PTKLNDLCFTNVYADSFVIRGNEVSQIAPGQTGNIADYNYKLPDDFTGCVIAWNSNKLDSKV<br>GGNYNYQYRLFRKSNLKPFERDISTEIYQAGNKPCNGVAGFNCYFPLRSYGFRPTYGVGHQP<br>YRVVVLSFELLHAPATVCGPKKSTNLVKNKCVNFNFNGLTGTGVLTESNKKFLPFQQFGRDIA<br>DTTDAVRDPQTLEILDITPCSFGGVSVITPGTNTSNQVAVLYQGVNCTEVPVAIHADQLTPTWR<br>VYSTGSNVFQTRAGCLIGAEYVNNSYECDIPIGAGICASYQTQTKSHRRARSVASQSIIAYTMS<br>LGAENLVAYSNNSIAIPTNFTISVTTEILPVSMTKTSVDCTMYICGDSTECSNLLLQYGSFCTQL<br>KRALTGIAVEQDKNTQEVFAQVKQIYKTPPIKYFGGFNFSQILPDPSKPSKRSFIEDLLFNKVTL

	ADAGFIKQYGDCLGDIAARDLICAQKFNGLTVLPPLLTDEMIAQYTSALLAGTITSGWTFGAG<br>AALQIPFAMQMAYRFNGIGVTQNVLYENQKLIANQFNSAIGKIQDSLSSTASALGKLQDVVN<br>HNAQALNTLVKQLSSKFGAISSVLNDILSRLDKVEAEVQIDRLITGRLQSLQTYVTQQLIRAA<br>EIRASANLAATKMSECVLGQSKRVDFCGKGYHLMSFPQSAPHGVVFLHVTYVPAQEKNFTT<br>APAICHDGKAHFPREGVFVSNGTHWFVTQRNFYEPQIITTDNTFVSGNCDVVIGIVNNTVYDP<br>LQPELDSFKEELDKYFKNHTSPDVDLGDISGINASVVNIQKEIDRLNEVAKNLNESLIDLQELG<br>KYEQYIKWPWYIWLGFIAGLIAIVMVTIMLCCMTSCCSCLKGCCSCGSCC
**BA.2.75**	MFVFLVLLPLVSSQCVNLITRTQSYTNSFTRGVYYPDKVFRSSVLHSTQDLFLPFFSNVTWFH<br>AIHVSGTNGTKRFDNPVLPFNDGVYFASTEKSNIIRGWIFGTTLDSKTQSLLIVNNATNVVIKV<br>CEFQFCNDPFLDVYYHENNKSRMESELRVYSSANNCTFEYVSQPFLMDLEGKQGNFKNLRE<br>FVFKNIDGYFKIYSKHTPVNLGRDLPQGFSALEPLVDLPIGINITRFQTLLALHRSYLTPGDSSS<br>SWTAGAAAYYVGYLQPRTFLLKYNENGTITDAVDCALDPLSETKCTLKSFTVEKGIYQTSNF<br>RVQPTESIVRFPNITNLCPFHEVFNATTFASVYAWNRKRISNCVADYSVLYNFAPFFAFKCYGV<br>SPTKLNDLCFTNVYADSFVIRGNEVSQIAPGQTGNIADYNYKLPDDFTGCVIAWNSNKLDSK<br>VSGNYNYLYRLFRKSKLKPFERDISTEIYQAGNKPCNGVAGSNCYFPLQSYGFRPTYGVGHQ<br>PYRVVVLSFELLHAPATVCGPKKSTNLVKNKCVNFNFNGLTGTGVLTESNKKFLPFQQFGRDI<br>ADTTDAVRDPQTLEILDITPCSFGGVSVITPGTNTSNQVAVLYQGVNCTEVPVAIHADQLTPTW<br>RVYSTGSNVFQTRAGCLIGAEYVNNSYECDIPIGAGICASYQTQTKSHRRARSVASQSIIAYTM<br>SLGAENSVAYSNNSIAIPTNFTISVTTEILPVSMTKTSVDCTMYICGDSTECSNLLLQYGSFCTQ<br>LKRALTGIAVEQDKNTQEVFAQVKQIYKTPPIKYFGGFNFSQILPDPSKPSKRSFIEDLLFNKVT<br>LADAGFIKQYGDCLGDIAARDLICAQKFNGLTVLPPLLTDEMIAQYTSALLAGTITSGWTFGA<br>GAALQIPFAMQMAYRFNGIGVTQNVLYENQKLIANQFNSAIGKIQDSLSSTASALGKLQDVV<br>NHNAQALNTLVKQLSSKFGAISSVLNDILSRLDKVEAEVQIDRLITGRLQSLQTYVTQQLIRA<br>AEIRASANLAATKMSECVLGQSKRVDFCGKGYHLMSFPQSAPHGVVFLHVTYVPAQEKNFT<br>TAPAICHDGKAHFPREGVFVSNGTHWFVTQRNFYEPQIITTDNTFVSGNCDVVIGIVNNTVYD<br>PLQPELDSFKEELDKYFKNHTSPDVDLGDISGINASVVNIQKEIDRLNEVARNLNESLINLQEL<br>GKYEQYIKWPWYIWLGFIAGLIAIVMVTIMLCCMTSCCSCLKGCCSCGSCC
**BA.5**	MFVFLVLLPLVSSQCVNLITRTQSYTNSFTRGVYYPDKVFRSSVLHSTQDLFLPFFSNVTWFH<br>AISGTNGTKRFDNPVLPFNDGVYFASTEKSNIIRGWIFGTTLDSKTQSLLIVNNATNVVIKVCE<br>FQFCNDPFLDVYYHKNNKSWMESEFRVYSSANNCTFEYVSQPFLMDLEGKQGNFKNLREFV<br>FKNIDGYFKIYSKHTPINLGRDLPQGFSALEPLVDLPIGINITRFQTLLALHRSYLTPGDSSSGW<br>TAGAAAYYVGYLQPRTFLLKYNENGTITDAVDCALDPLSETKCTLKSFTVEKGIYQTSNFRV<br>QPTESIVRFPNITNLCPFDEVFNATRFASVYAWNRKRISNCVADYSVLYNFAPFFAFKCYGVSP<br>TKLNDLCFTNVYADSFVIRGNEVSQIAPGQTGNIADYNYKLPDDFTGCVIAWNSNKLDSKVG<br>GNYNYRYRLFRKSNLKPFERDISTEIYQAGNKPCNGVAGVNCYFPLQSYGFRPTYGVGHQPY<br>RVVVLSFELLHAPATVCGPKKSTNLVKNKCVNFNFNGLTGTGVLTESNKKFLPFQQFGRDIAD<br>TTDAVRDPQTLEILDITPCSFGGVSVITPGTNTSNQVAVLYQGVNCTEVPVAIHADQLTPTWRV<br>YSTGSNVFQTRAGCLIGAEYVNNSYECDIPIGAGICASYQTQTKSHRRARSVASQSIIAYTMSL<br>GAENSVAYSNNSIAIPTNFTISVTTEILPVSMTKTSVDCTMYICGDSTECSNLLLQYGSFCTQL<br>KRALTGIAVEQDKNTQEVFAQVKQIYKTPPIKYFGGFNFSQILPDPSKPSKRSFIEDLLFNKVTL<br>ADAGFIKQYGDCLGDIAARDLICAQKFNGLTVLPPLLTDEMIAQYTSALLAGTITSGWTFGAG<br>AALQIPFAMQMAYRFNGIGVTQNVLYENQKLIANQFNSAIGKIQDSLSSTASALGKLQDVVN<br>HNAQALNTLVKQLSSKFGAISSVLNDILSRLDKVEAEVQIDRLITGRLQSLQTYVTQQLIRAA<br>EIRASANLAATKMSECVLGQSKRVDFCGKGYHLMSFPQSAPHGVVFLHVTYVPAQEKNFTT

	APAICHDGKAHFPREGVFVSNGTHWFVTQRNFYEPQIITTDNTFVSGNCDVVIGIVNNTVYDP<br>LQPELDSFKEELDKYFKNHTSPDVDLGDISGINASVVNIQKEIDRLNEVAKNLNESLIDLQELG<br>KYEQYIKWPWYIWLGFIAGLIAIVMVTIMLCCMTSCCSCLKGCCSCGSCC
**BF.7**	MFVFLVLLPLVSSQCVNLITRTQSYTNSFTRGVYYPDKVFRSSVLHSTQDLFLPFFSNVTWFH<br>AISGTNGTKRFDNPVLPFNDGVYFASTEKSNIIRGWIFGTTLDSKTQSLLIVNNATNVVIKVCE<br>FQFCNDPFLDVYYHKNNKSWMESEFRVYSSANNCTFEYVSQPFLMDLEGKQGNFKNLREFV<br>FKNIDGYFKIYSKHTPINLGRDLPQGFSALEPLVDLPIGINITRFQTLLALHRSYLTPGDSSSGW<br>TAGAAAYYVGYLQPRTFLLKYNENGTITDAVDCALDPLSETKCTLKSFTVEKGIYQTSNFRV<br>QPTESIVRFPNITNLCPFDEVFNATTFASVYAWNRKRISNCVADYSVLYNFAPFFAFKCYGVSP<br>TKLNDLCFTNVYADSFVIRGNEVSQIAPGQTGNIADYNYKLPDDFTGCVIAWNSNKLDSKVG<br>GNYNYRYRLFRKSNLKPFERDISTEIYQAGNKPCNGVAGVNCYFPLQSYGFRPTYGVGHQPY<br>RVVVLSFELLHAPATVCGPKKSTNLVKNKCVNFNFNGLTGTGVLTESNKKFLPFQQFGRDIAD<br>TTDAVRDPQTLEILDITPCSFGGVSVITPGTNTSNQVAVLYQGVNCTEVPVAIHADQLTPTWRV<br>YSTGSNVFQTRAGCLIGAEYVNNSYECDIPIGAGICASYQTQTKSHRRARSVASQSIIAYTMSL<br>GAENSVAYSNNSIAIPTNFTISVTTEILPVSMTKTSVDCTMYICGDSTECSNLLLQYGSFCTQL<br>KRALTGIAVEQDKNTQEVFAQVKQIYKTPPIKYFGGFNFSQILPDPSKPSKRSFIEDLLFNKVTL<br>ADAGFIKQYGDCLGDIAARDLICAQKFNGLTVLPPLLTDEMIAQYTSALLAGTITSGWTFGAG<br>AALQIPFAMQMAYRFNGIGVTQNVLYENQKLIANQFNSAIGKIQDSLSSTASALGKLQDVVN<br>HNAQALNTLVKQLSSKFGAISSVLNDILSRLDKVEAEVQIDRLITGRLQSLQTYVTQQLIRAA<br>EIRASANLAATKMSECVLGQSKRVDFCGKGYHLMSFPQSAPHGVVFLHVTYVPAQEKNFTT<br>APAICHDGKAHFPREGVFVSNGTHWFVTQRNFYEPQIITTDNTFVSGNCDVVIGIVNNTVYDP<br>LQPELDSFKEELDKYFKNHTSPDVDLGDISGINASVVNIQKEIDRLNEVAKNLNESLIDLQELG<br>KYEQYIKWPWYIWLGFIAGLIAIVMVTIMLCCMTSCCSCLKGCCSCGSCC
**BQ.1.1**	MFVFLVLLPLVSSQCVNLTTRTQLPPAYTNSFTRGVYYPDKVFRSSVLHSTQDLFLPFFSNVT<br>WFHAISGTNGTKRFDNPVLPFNDGVYFASTEKSNIIRGWIFGTTLDSKTQSLLIVNNATNVVIK<br>VCEFQFCNDPFLDVYYHKNNKSWMESEFRVYSSANNCTFEYVSQPFLMDLEGKQGNFKNL<br>REFVFKNIDGYFKIYSKHTPINLGRDLPQGFSALEPLVDLPIGINITRFQTLLALHRSYLTPGDSS<br>SGWTAGAAAYYVGYLQPRTFLLKYNENGTITDAVDCALDPLSETKCTLKSFTVEKGIYQTSN<br>FRVQPTESIVRFPNITNLCPFDEVFNATTFASVYAWNRKRISNCVADYSVLYNFAPFFAFKCYG<br>VSPTKLNDLCFTNVYADSFVIRGNEVSQIAPGQTGNIADYNYKLPDDFTGCVIAWNSNKLDS<br>TVGGNYNYRYRLFRKSKLKPFERDISTEIYQAGNKPCNGVAGVNCYFPLQSYGFRPTYGVGH<br>QPYRVVVLSFELLHAPATVCGPKKSTNLVKNKCVNFNFNGLTGTGVLTESNKKFLPFQQFGR<br>DIADTTDAVRDPQTLEILDITPCSFGGVSVITPGTNTSNQVAVLYQGVNCTEVPVAIHADQLTP<br>TWRVYSTGSNVFQTRAGCLIGAEYVNNSYECDIPIGAGICASYQTQTKSHRRARSVASQSIIA<br>YTMSLGAENSVAYSNNSIAIPTNFTISVTTEILPVSMTKTSVDCTMYICGDSTECSNLLLQYGS<br>FCTQLKRALTGIAVEQDKNTQEVFAQVKQIYKTPPIKYFGGFNFSQILPDPSKPSKRSFIEDLLF<br>NKVTLADAGFIKQYGDCLGDIAARDLICAQKFNGLTVLPPLLTDEMIAQYTSALLAGTITSG<br>WTFGAGAALQIPFAMQMAYRFNGIGVTQNVLYENQKLIANQFNSAIGKIQDSLSSTASALGK<br>LQDVVNHNAQALNTLVKQLSSKFGAISSVLNDILSRLDKVEAEVQIDRLITGRLQSLQTYVTQ<br>QLIRAAEIRASANLAATKMSECVLGQSKRVDFCGKGYHLMSFPQSAPHGVVFLHVTYVPAQ<br>EKNFTTAPAICHDGKAHFPREGVFVSNGTHWFVTQRNFYEPQIITTDNTFVSGNCDVVIGIVN<br>NTVYDPLQPELDSFKEELDKYFKNHTSPDVDLGDISGINASVVNIQKEIDRLNEVAKNLNESLI<br>DLQELGKYEQYIKWPWYIWLGFIAGLIAIVMVTIMLCCMTSCCSCLKGCCSCGSCC
**XBB.1.5**	MFVFLVLLPLVSSQCVNLITRTQSYTNSFTRGVYYPDKVFRSSVLHSTQDLFLPFFSNVTWFH

	AIHVSGTNGTKRFDNPALPFNDGVYFASTEKSNIIRGWIFGTTLDSKTQSLLIVNNATNVVIKV<br>CEFQFCNDPFLDVYQKNNKSWMESEFRVYSSANNCTFEYVSQPFLMDLEGKEGNFKNLREF<br>VFKNIDGYFKIYSKHTPINLERDLPQGFSALEPLVDLPIGINITRFQTLLALHRSYLTPVDSSSG<br>WTAGAAAYYVGYLQPRTFLLKYNENGTITDAVDCALDPLSETKCTLKSFTVEKGIYQTSNFR<br>VQPTESIVRFPNITNLCPFHEVFNATTFASVYAWNRKRISNCVADYSVIYNFAPFFAFKCYGVS<br>PTKLNDLCFTNVYADSFVIRGNEVSQIAPGQTGNIADYNYKLPDDFTGCVIAWNSNKLDSKP<br>SGNYNYLYRLFRKSKLKPFERDISTEIYQAGNKPCNGVAGPNCYSPLQSYGFRPTYGVGHQP<br>YRVVVLSFELLHAPATVCGPKKSTNLVKNKCVNFNFNGLTGTGVLTESNKKFLPFQQFGRDIA<br>DTTDAVRDPQTLEILDITPCSFGGVSVITPGTNTSNQVAVLYQGVNCTEVPVAIHADQLTPTWR<br>VYSTGSNVFQTRAGCLIGAEYVNNSYECDIPIGAGICASYQTQTKSHRRARSVASQSIIAYTMS<br>LGAENSVAYSNNSIAIPTNFTISVTTEILPVSMTKTSVDCTMYICGDSTECSNLLLQYGSFCTQL<br>KRALTGIAVEQDKNTQEVFAQVKQIYKTPPIKYFGGFNFSQILPDPSKPSKRSFIEDLLFNKVTL<br>ADAGFIKQYGDCLGDIAARDLICAQKFNGLTVLPPLLTDEMIAQYTSALLAGTITSGWTFGAG<br>AALQIPFAMQMAYRFNGIGVTQNVLYENQKLIANQFNSAIGKIQDSLSSTASALGKLQDVVN<br>HNAQALNTLVKQLSSKFGAISSVLNDILSRLDKVEAEVQIDRLITGRLQSLQTYVTQQLIRAA<br>EIRASANLAATKMSECVLGQSKRVDFCGKGYHLMSFPQSAPHGVVFLHVTYVPAQEKNFTT<br>APAICHDGKAHFPREGVFVSNGTHWFVTQRNFYEPQIITTDNTFVSGNCDVVIGIVNNTVYDP<br>LQPELDSFKEELDKYFKNHTSPDVDLGDISGINASVVNIQKEIDRLNEVAKNLNESLIDLQELG<br>KYEQYIKWPWYIWLGFIAGLIAIVMVTIMLCCMTSCCSCLKGCCSCGSCC
**EG.5**	MFVFLVLLPLVSSQCVNLITRTQSYTNSFTRGVYYPDKVFRSSVLHSTQDLFLPFFSNVTWFH<br>AIHVSGTNGTKRFDNPALPFNDGVYFASTEKSNIIRGWIFGTTLDSKTQSLLIVNNATNVVIKV<br>CEFQFCNDPFLDVYQKNNKSWMESEFRVYSSANNCTFEYVSQPFLMDLEGKEGNFKNLREF<br>VFKNIDGYFKIYSKHTPINLERDLPQGFSALEPLVDLPIGINITRFQTLLALHRSYLTPVDSSSG<br>WTAGAAAYYVGYLQPRTFLLKYNENGTITDAVDCALDPLSETKCTLKSFTVEKGIYQTSNFR<br>VQPTESIVRFPNITNLCPFHEVFNATTFASVYAWNRKRISNCVADYSVIYNFAPFFAFKCYGVS<br>PTKLNDLCFTNVYADSFVIRGNEVSQIAPGQTGNIADYNYKLPDDFTGCVIAWNSNKLDSKP<br>SGNYNYLYRLLRKSKLKPFERDISTEIYQAGNKPCNGVAGPNCYSPLQSYGFRPTYGVGHQP<br>YRVVVLSFELLHAPATVCGPKKSTNLVKNKCVNFNFNGLTGTGVLTESNKKFLPFQQFGRDIA<br>DTTDAVRDPQTLEILDITPCSFGGVSVITPGTNTSNQVAVLYQGVNCTEVPVAIHADQLTPTWR<br>VYSTGSNVFQTRAGCLIGAEYVNNSYECDIPIGAGICASYQTQTKSHRRARSVASQSIIAYTMS<br>LGAENLVAYSNNSIAIPTNFTISVTTEILPVSMTKTSVDCTMYICGDSTECSNLLLQYGSFCTQL<br>KRALTGIAVEQDKNTQEVFAQVKQIYKTPPIKYFGGFNFSQILPDPSKPSKRSFIEDLLFNKVTL<br>ADAGFIKQYGDCLGDIAARDLICAQKFNGLTVLPPLLTDEMIAQYTSALLAGTITSGWTFGAG<br>AALQIPFAMQMAYRFNGIGVTQNVLYENQKLIANQFNSAIGKIQDSLSSTASALGKLQDVVN<br>HNAQALNTLVKQLSSKFGAISSVLNDILSRLDKVEAEVQIDRLITGRLQSLQTYVTQQLIRAA<br>EIRASANLAATKMSECVLGQSKRVDFCGKGYHLMSFPQSAPHGVVFLHVTYVPAQEKNFTT<br>APAICHDGKAHFPREGVFVSNGTHWFVTQRNFYEPQIITTDNTFVSGNCDVVIGIVNNTVYDP<br>LQPELDSFKEELDKYFKNYTSPDVDLGDISGINASVVNIQKEIDRLNEVAKNLNESLIDLQELG<br>KYEQYIKWPWYIWLGFIAGLIAIVMVTIMLCCMTSCCSCLKGCCSCGSCC
